# Distinguishing *Leptothrix* and *Sphaerotilus* genera by an integrated genomic-phenotypic analysis supported by new *Leptothrix* genomes

**DOI:** 10.1128/msystems.01768-25

**Published:** 2026-06-12

**Authors:** Gracee K. Tothero, Jessica L. Keffer, David Emerson, Emily J. Fleming, Clara S. Chan

**Affiliations:** 1 Delaware Biotechnology Institute, Newark, Delaware, USA; 2 Department of Earth Sciences, University of Delaware 5972, Newark, Delaware, USA; 3 Microbiology Graduate Program, University of Delaware 5972, Newark, Delaware, USA; 4 Bigelow Laboratory for Ocean Sciences 8506, East Boothbay, Maine, USA; 5 Department of Biological Sciences, California State University 14663, Chico, California, USA; University of Wisconsin-Milwaukee, Milwaukee, Wisconsin, USA

**Keywords:** iron oxidation, *Leptothrix*, *Sphaerotilus*, pangenome, metagenomics

## Abstract

The *Sphaerotilus-Leptothrix* group of bacteria includes one of the first described microorganisms, *Leptothrix ochracea*, an uncultured type strain, plus isolates of *Leptothrix* and *Sphaerotilus*. This group is unified by the ability to form sheaths and oxidize metals, although *L. ochracea* exhibits obvious ecological, morphological, and functional differences from the rest of *Sphaerotilus-Leptothrix*. Recently, there have been calls to combine the group into one genus, *Sphaerotilus*; however, these studies lacked adequate genomic representation of *L. ochracea*. Here, we present a comprehensive comparative genomic analysis of the *Sphaerotilus-Leptothrix* group, including expanded representation of *L. ochracea*, a closely related novel species, *Leptothrix toolikensis*, and two new isolates (*Leptothrix mechoopdaensis*). Analysis of 38 genomes resolves three phylogenetic and functional groups: the *ochracea*-type *Leptothrix* (Group 1), the *mobilis*-type *Leptothrix* (Group 2), and *Sphaerotilus* (Group 3). Group 1 genomes form a separate genus based on average nucleotide identity and alignment fraction. The genomes clearly diverge from the rest of *Sphaerotilus-Leptothrix* in phylogeny, size, and metabolic potential. Group 1 genomes are much smaller (2.59–3.04 Mb) than those of Groups 2 (4.55–6.06 Mb) and 3 (3.94–5.07 Mb), while encoding more metal oxidases and fewer carbohydrate-active enzymes. Group 2 clusters with Group 3 phylogenetically and is similar in organic carbon metabolisms but maintains more metal oxidation genes. Group 2 members lack homogeneity in phenotype and genotype, suggesting that additional isolates and genomes are needed for confident classification. However, Group 1 genomes (*L. ochracea* and *L. toolikensis*) show clear divergence, precluding their inclusion in *Sphaerotilus* and supporting the retention of the genus *Leptothrix*.

**IMPORTANCE:**

Researchers have long noted differences in metal oxidation, morphology, and ecology among *Sphaerotilus-Leptothrix*, but longstanding confusion over phylogeny and genus boundaries led to inconsistent taxonomic classification between the two genera. This confusion stems from previous work that used isolates that are unavailable or lost distinguishing traits in culture, and from limited genomic data. Furthermore, the *Leptothrix* type strain *L. ochracea* has never been isolated. This study provides molecular evidence that substantiates calls to reassign some *Leptothrix* members to the genus *Sphaerotilus* but adds to an emerging body of evidence that Group 1 *L. ochracea* and now *L. toolikensis* represent a functionally distinct lineage. While genomic similarity metrics left taxonomic divisions unclear, integrating metabolic potential with phylogeny resolved genus boundaries based on clear functional groupings. This polyphasic approach for delineating genera clarifies longstanding taxonomic confusion and refines our understanding of functional diversity both across and within *Sphaerotilus-Leptothrix* lineages.

## INTRODUCTION

The bacteria of the *Sphaerotilus-Leptothrix* group (SLG) are among the earliest described microbes due to their formation of clearly identifiable filaments that form biofilms, flocs, and mats. Studies on SLG date back to 1797 (1–5) and have historically considered *Leptothrix* and *Sphaerotilus* close relatives based on morphological and physiological traits (6–10). Both genera are metal-oxidizing bacteria that exhibit filamentous growth; chains of cells are enclosed in tubular sheaths, which can become encrusted in iron and/or manganese oxides (4–7, 11–18). The close association of *Leptothrix* and *Sphaerotilus* proved justified by phylogenetic analysis, initially of 16S rRNA and *mofA* genes, and most recently whole genome comparisons (6, 19–26). In fact, by some metrics, genomic analysis suggests that the group should be a single genus (26, 27). However, *L. ochracea* has always stood out as an outlier in its preference for low organic carbon environments and its prodigious production of iron-mineralized sheaths.

*Leptothrix ochracea* was the first described member of SLG (1) and remains the type species of *Leptothrix*; however*,* it has never been isolated, making it difficult to study its physiology and niche. Instead, isolates of *Leptothrix* and *Sphaerotilus* have served as models for describing this group. All SLG isolates grow organoheterotrophically (7, 17, 18, 28, 29); *Sphaerotilus* is found in environments with higher organic carbon (wastewater, organic-polluted streams) and its growth responds more strongly to organics, while most *Leptothrix* respond minimally, with the exception of *L. cholodnii* (6). Both *Leptothrix* and *Sphaerotilus* can oxidize iron, but *Sphaerotilus* do not tend to accumulate metal oxides in the environment like *Leptothrix* (6, 30). Manganese oxidation is generally considered unique to *Leptothrix* (6, 10), and while Schmidt et al. (24) reported that a *Sphaerotilus* isolate oxidized manganese, the ability to oxidize manganese is still considered a primary method for distinguishing *Leptothrix* from *Sphaerotilus*. Our understanding of the SLG is based on a limited number of isolates; thus, a more comprehensive functional analysis across a greater diversity of the group is still required to understand true distinctions in metal oxidation and organic utilization.

Phenotypic and ecological traits clearly distinguish the original, type species *L. ochracea* from other members of *Leptothrix*. Its sheaths become heavily encrusted in iron oxides and are mostly empty, containing few cells, unlike the sheaths of isolate species, which typically are densely populated with cells (6, 7, 12, 21, 31, 32). This growth pattern suggests that *L. ochracea* must oxidize extensive amounts of iron to meet its energetic needs, consistent with the low energy yield from iron oxidation (2, 12, 33–36). Xenic cultures can grow in Fe(II)-amended natural waters without any added organics and, in fact, require Fe(II) for growth (33). Genomic analyses give further support for iron oxidation as the major energy source and also for a mixotrophic metabolism with limited organic utilization abilities, in contrast to the broader substrate utilization capabilities of its organoheterotrophic relatives (21). These contrasts in function and genomic content suggest that *L. ochracea* differs substantially from other SLG in its impact on ecology and biogeochemistry, and the combined differences in phenotype, genotype, and ecotype could merit a separate genus.

Proper genus classification is significant because genera assignments are most commonly used for interpreting physiology, metabolism, and niche. For most of their history, *Leptothrix* and *Sphaerotilus* have been distinguished from each other and from close relatives based on phenotypes: cell size, sheath morphology, iron and manganese oxidation capabilities, organic carbon substrate utilization, and response to organics (6, 7, 10, 12, 24, 37). However, there have been numerous classification mistakes due to the loss of phenotypes in cultures post-isolation (e.g., sheaths and Mn oxidation) or phenotypic variations between culture conditions (6, 8, 9, 12, 24, 38–40). This led to a convoluted history of renaming between the two genera (7, 12, 34, 40). The confusion between genera was not completely alleviated once phylogenetic options became available, since *Leptothrix* and *Sphaerotilus* are closely related to each other and to other members of the Sphaerotilaceae (19, 36, 41–45). Consequently, it is often unclear whether an organism belongs to *Leptothrix* or *Sphaerotilus*, even when molecular data are available. These difficulties are perpetuated by incorrect and incomplete classifications of SLG in reference databases (e.g., Silva; see Tothero et al. [21]). Stable phylogenies depend on sufficient numbers of sequences with relatively even distributions across each clade. Prior genome-based reclassification efforts relied on a sparse subset of reference genomes that either missed *L. ochracea* or included only partial representation of *Leptothrix* (26, 27, 44). A fair, consistent evaluation of the *Leptothrix* and *Sphaerotilus* genera requires the use of a robust molecular data set with adequate reference sequences, including representation from more than one *L. ochracea* genome.

To better resolve phylogenetic relationships and characterize functional diversity within the group, we performed a comprehensive comparative analysis of 38 high-quality genomes of *Sphaerotilus-Leptothrix* members. These include new genomes that fill in missing diversity in *Leptothrix* with two novel species represented by four metagenome-assembled genomes (*Leptothrix toolikensis*) and two isolate genomes (*Leptothrix mechoopdaensis*). We used the full set of sequences to delineate three distinct subgroups within the SLG: the *ochracea*-type *Leptothrix* (Group 1), the *mobilis*-type *Leptothrix* (Group 2), and *Sphaerotilus* (Group 3). We searched for genes indicating key metabolisms known to distinguish *Leptothrix* from *Sphaerotilus*, including metal oxidation, carbon fixation, and organic carbon utilization. This work establishes a framework and a comprehensive reference data set with which to identify additional SLG by sequence-based methods.

## RESULTS

### Phylogeny and genome features

**Fig 1:**
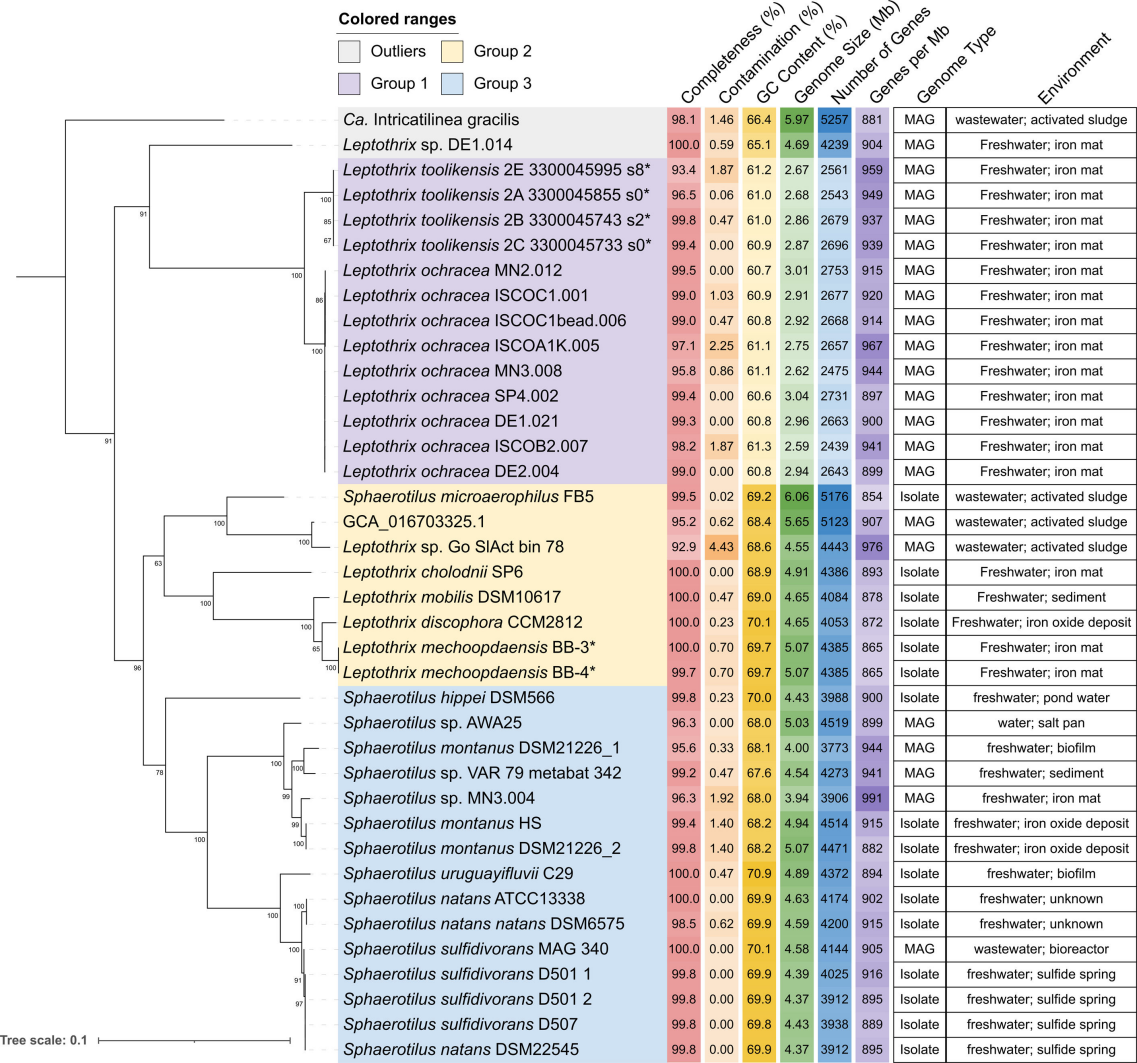
Phylogeny of the *Sphaerotilus-Leptothrix* group. Maximum likelihood tree of concatenated ribosomal proteins from genomes in SLG, using *Rhodoferax ferrireducens* T118 as an outgroup (not shown). Bootstrap support between 50% and 100% shown. Generated using RAxML with 1,000 bootstraps from alignment of 39 ribosomal proteins. *, newly sequenced genomes.

We collected 38 high-quality SLG genomes, which include 16 isolates and 22 metagenome-assembled genomes (MAGs) from our own work and public databases (Table S1; Supplemental text), employing cutoffs of 90% completeness and 5% contamination, in accordance with MIMAG standards for high-quality draft genomes (46). This includes four new MAGs from Arctic iron microbial mats (see Michaud et al. [47] for site information), and two newly sequenced genomes of isolates (*Leptothrix mechoopdaensis*) from temperate stream iron mats (Fleming et al., [48]). Genomes were classified as SLG based on clustering in a monophyletic clade with known SLG in a concatenated ribosomal protein tree (Fig. 1).

**Fig 2:**
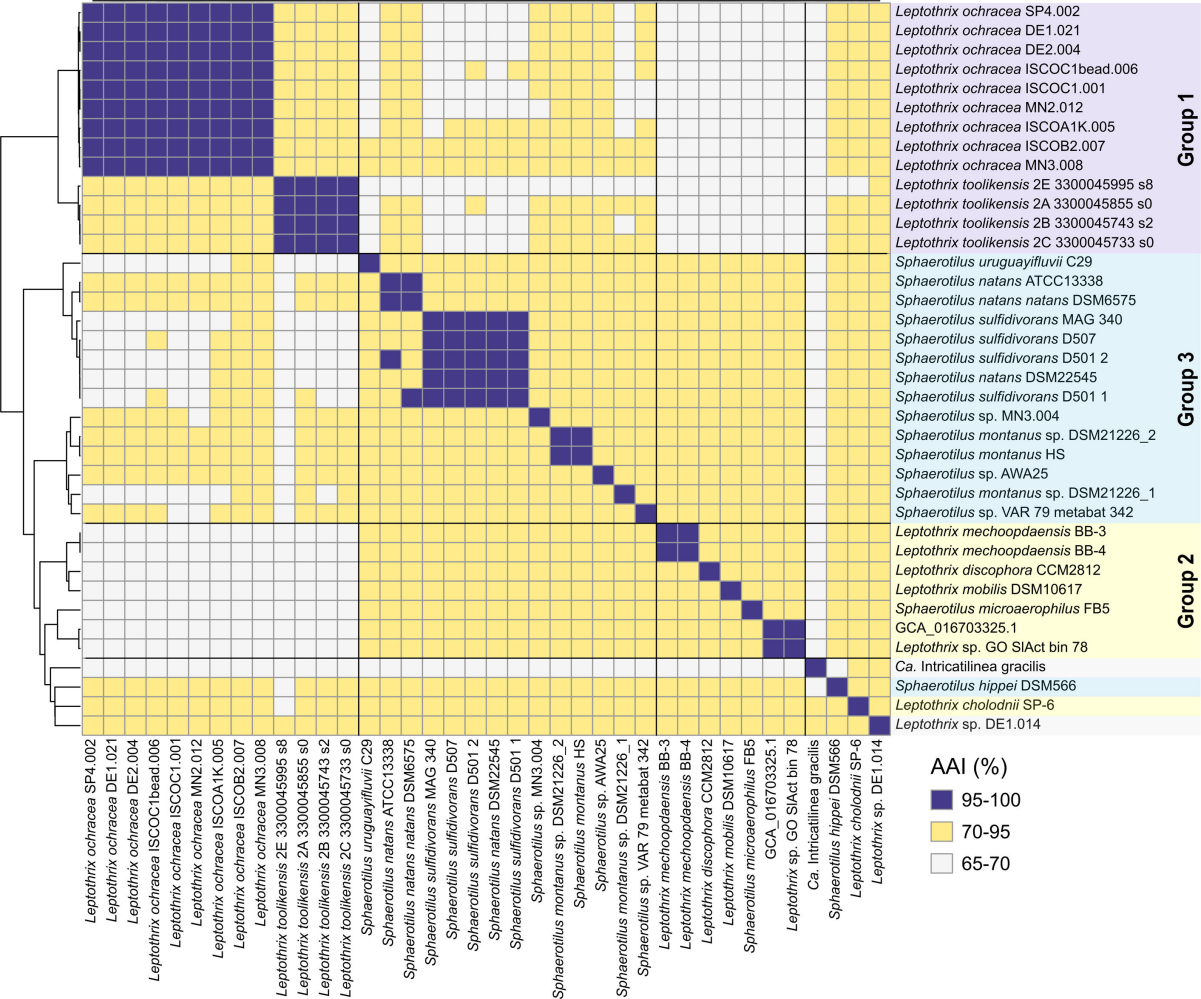
Amino acid identity (%) between SLG genomes. AAI values colored by species-level (95%, blue) and genus-level (70%, yellow; 65%, gray) cutoffs.

Concatenated trees revealed three major subgroups within SLG; these groups cluster with *Leptothrix ochracea*, *Leptothrix mobilis*, and *Sphaerotilus natans* (Fig. 1; Fig. S1). We designate these three subgroups as SLG Group 1 (the *ochracea*-type *Leptothrix*), SLG Group 2 (the *mobilis*-type *Leptothrix*), and SLG Group 3 (*Sphaerotilus*). Despite their historical placement in different genera, the *L. mobilis* clade clusters more closely to the *S. natans* clade than it does to *L. ochracea* with high bootstrap support (91%–96%). Two genomes clustered with SLG but emerged as outliers (Fig. 1; Fig. S1); these include *Leptothrix* DE1.014 and one genome that was previously reclassified from *Leptothrix* to “*Candidatus* Intricatilinea gracilis” (44). However, “*Ca*. I. gracilis” exhibits the filamentous morphology characteristic of *Leptothrix* (44), supporting the inclusion of “*Ca*. I. gracilis” in this analysis. Amino acid identity (AAI) analysis of the two outliers showed they are sufficiently close (>70% identity) to *L. cholodnii* SP-6 to be considered part of the same genus (Fig. 2; [49]). Consequently, they are included but were not assigned to a specific subgroup.

AAI and average nucleotide identity (ANI) metrics can help estimate the species-level diversity among the three groups. Based on the thresholds of 95% AAI and ANI (50, 51), Group 1 contains two SLG species, Group 2 has six, and Group 3 has nine (Fig. 2; Table S2). The majority of Group 2 and some of the Group 3 genomes are single representatives of their own species. While these AAI- and ANI-based patterns reflect considerable diversity across Groups 2 and 3, they also suggest that sequencing efforts have not yet saturated the full diversity of SLG. Group 2 includes two genomes of novel *Leptothrix* isolates, *Leptothrix mechoopdaensis* strains BB-3 and BB-4, which were isolated from an iron seep in Centerville, CA (as described by Fleming et al. [48]). They are most closely related to *L. discophora* but do not meet the 95% AAI or ANI cutoff to be considered the same species (AAI = 87.7%; ANI = 89.4%). Therefore, we designate these isolates a novel *Leptothrix* species, *L. mechoopdaensis*. Group 1 genomes include *L. ochracea* (21), and four newly sequenced MAGs from an Arctic tundra iron seep that are most closely related to *L. ochracea*, but cluster separately in the tree and do not meet the 95% AAI or ANI cutoff to be considered the same species as *L. ochracea* (AAI = 90.0%–90.4%; ANI = 90.0%–90.1%). Therefore, we designate these MAGs a novel *Leptothrix* species, *L. toolikensis*. This species represents the first sequenced species that is closely related to, yet separate from, *L. ochracea*.

Using AAI metrics, it is not completely clear if Groups 1, 2, and 3 represent one or two genera. Genus-level AAI thresholds are not well agreed upon but generally range from 65% to 70% (27, 49). If the most inclusive genus-level AAI threshold proposed for the family Sphaerotilaceae is used (67.12% [27]), all high-quality genomes of SLG analyzed could belong to a single genus (Fig. 2). Using the strictest cutoff of 70% does not delineate clear boundaries between Groups 2 and 3; all representatives from Groups 2 and 3 could be considered the same genus. On the other hand, the Group 1 genomes meet this threshold with a minority of genomes from Groups 2 and 3. Thus, it is not clear based on AAI alone that *L. ochracea* belongs to the same genus as other SLG.

The percentage of conserved proteins (POCP) can be used as an additional metric for genus delineation at a threshold of 50% (52, 53). Similar to AAI, the genera *Leptothrix* and *Sphaerotilus* cannot be divided cleanly using this threshold (Fig. S2). However, Group 1 genomes clearly show high POCP using this metric, and cluster separately from Groups 2 and 3, adding to evidence of the distinctiveness of Group 1.

**Fig 3:**
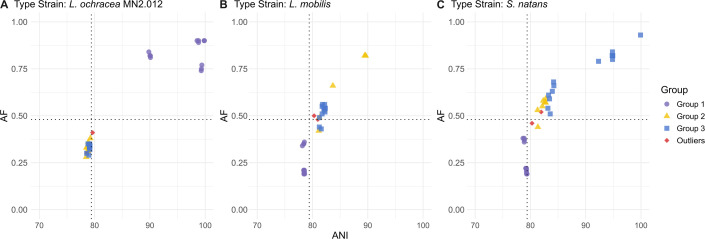
Average nucleotide identity (ANI) versus alignment fraction (AF) for all genomes in this study, compared to each of the three type strains. (**A**) *L. ochracea* MN2.012, (**B**) *L. mobilis* DSM10617, (**C**) *S. natans* CCM2812. Each panel shows pairwise comparisons to the type strain. Dashed lines indicate genus-level thresholds of 79.40% ANI and 0.480 AF, averages of the thresholds for Burkholderiaceae and Comamonadaceae defined by Barco et al. (54). Calculated using ANIcalculator 2014-127, version 1.0 (https://ani.jgi.doe.gov/html/download.php).

Genomic similarity metrics can be combined in a method proposed by Barco et al. (54) to delineate genus-level boundaries based on ANI and alignment fraction (AF) to genus type strains. We applied this method using three type strains: the highest quality *L. ochracea* genome (MN2.012), *S. natans* ATCC13338, and *L. mobilis* DSM10617; we repeated this method using multiple ANI and AF calculation methods (Fig. 3; Fig. S3; Table S2). Since the thresholds have not been defined for the family Sphaerotilaceae, we used the averaged Burkholderiaceae and Comamonadaceae ratio (AF:ANI; 0.480:79.40; [54]). Based on this analysis, Group 1 is distinct from other SLG, but Groups 2 and 3 do not cluster separately from each other, consistent with the AAI and POCP analyses. Thus, various genomic metrics do not distinguish Group 2 *Leptothrix* and Group 3 *Sphaerotilus* as separate genera; however, Group 1 is clearly distinguishable.

Much like these sequence-based distinctions, genome size and GC content demonstrate that Group 1 is distinct from Groups 2 and 3 (Table 1; Table S1). While Group 1 genomes are 2.83 Mb on average, Groups 2 and 3 are 4.55 and 5.08 Mb on average, and there is no overlap between the genome size ranges of Group 1 and Groups 2 and 3. The number of genes per genome corresponds to these differences; Group 1 genomes are ~60% the size of the genomes in Groups 2 and 3 and contain ~60% the number of genes. Accordingly, the gene density in all three groups is roughly the same, with around 900 genes per Mb. In addition, the average GC content of Group 1 is 8.3% lower than that of Groups 2 and 3. These metrics support that Group 1 (*L. ochracea* and *L. toolikensis*) is distinct among SLG.

**TABLE 1:** Genome metrics across SLG subgroups

Group	Description	Avg size (Mb)	Size range (Mb)	Avg GC%	GC% range	Avg no. of genes	No. of genes (range)
1	*ochracea*-like *Leptothrix*	2.83	2.59–3.04	60.9	60.6–61.3	2,630	2,439–2,753
2	*mobilis*-like *Leptothrix*	5.08	4.55–6.06	69.2	68.4–70.1	4,504	4,053–5,176
3	*Sphaerotilus*	4.55	3.94–5.07	69.2	67.6–70.9	4,141	3,773–4519

### SLG pangenomic comparison

**Fig 4:**
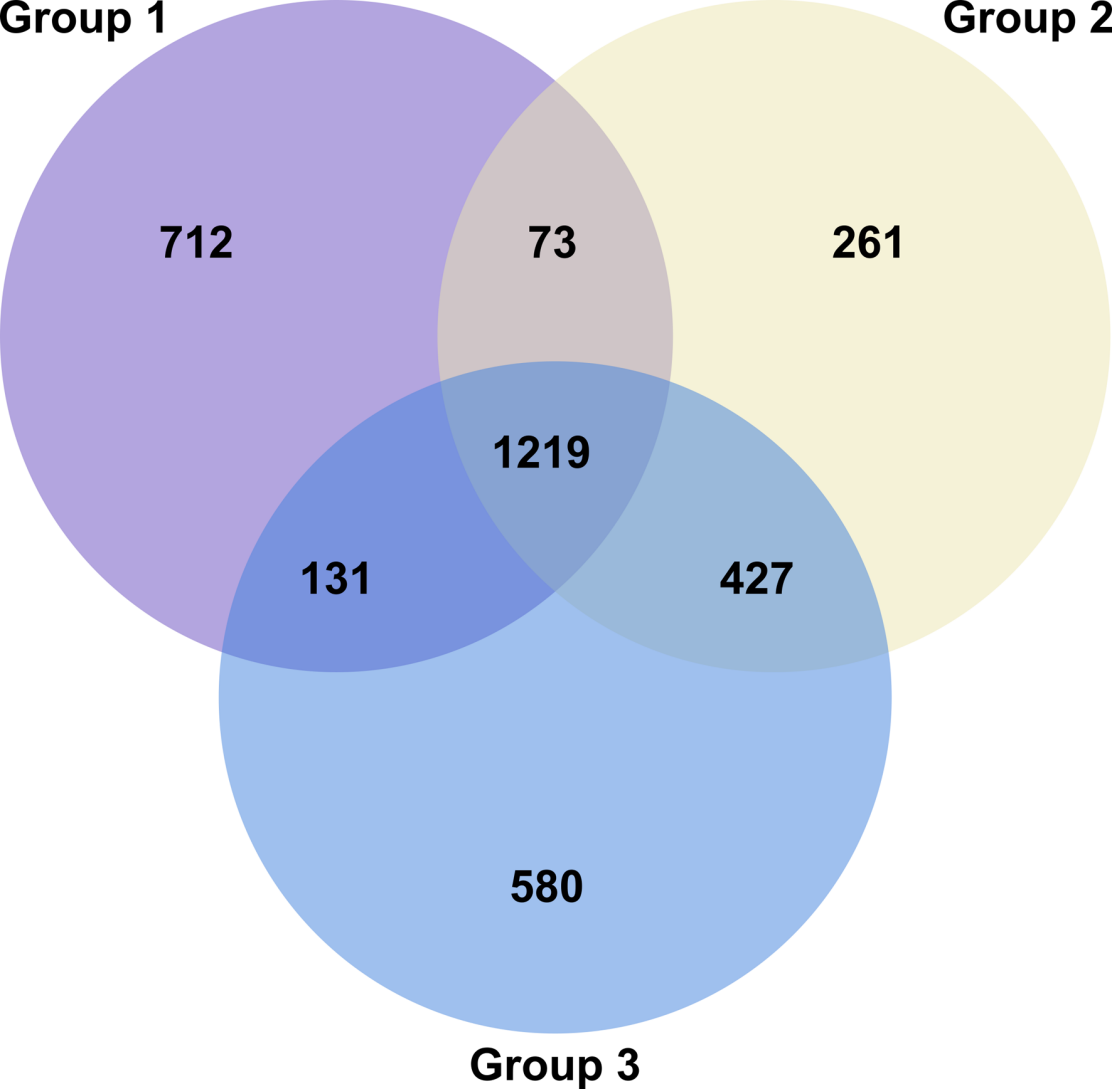
Venn diagram showing the distribution of near-core genes across SLG groups. Gene clusters are considered near-core if they are present in >85% of genomes in at least one SLG group.

Pangenomic comparisons can help reveal the extent of commonalities and differences among individual genomes and groups. Across 38 genomes, 20,884 gene clusters were identified, including 8,948 singleton gene clusters (43% of the clusters), that is, genes that occur in only one genome. Among Groups 1, 2, and 3 (excluding the outlier genomes), 18,214 gene clusters were identified, including 6,320 singletons (35% of the clusters) (Table S1), and 865 (4.7% of the clusters) genes that were shared by 100% of the genomes (core gene set). The genomes in this study are 90%–100% complete, so SLG likely contains an even greater number of shared gene clusters. To account for genes missing due to completeness, we designated near-core gene sets based on the presence of a gene cluster in at least 85% of each group of genomes (32 of 38; Fig. 4). The near-core gene set of SLG, excluding the outliers, contained 1,219 genes (6.4% of the clusters). Considering that SLG have 2,439–5,257 genes per genome, 23%–50% of each genome is composed of near-core genes. Furthermore, Groups 2 and 3 have more singleton gene clusters than Group 1 (Table S1), reflecting broader gene content differences among Groups 2 and 3, while Group 1 showed limited variation between strains. Group 1 had the largest number of gene clusters specific to its near-core gene set, despite having the smallest genomes, and shared few gene clusters exclusively with Group 2 or Group 3 (Fig. 4).

### Genomic potential

We conducted a comparative genomic analysis to explore differences in metabolic potential across the three groups. We identified key genes for energy generation (metal oxidation, organic carbon utilization, and oxidation of other inorganic electron donors) and biomass formation (organic carbon assimilation and CO_2_ fixation). In addition, we identified key genes for sheath formation. Genomic potential for oxygen reduction, vitamin transport, and polymer storage is described in the Supplemental text. A curated list of gene cluster frequencies and their functional annotations is provided in Table S3.

#### Energy generation by oxidation of metals and other inorganic electron donors

#### Iron oxidases

**Fig 5:**
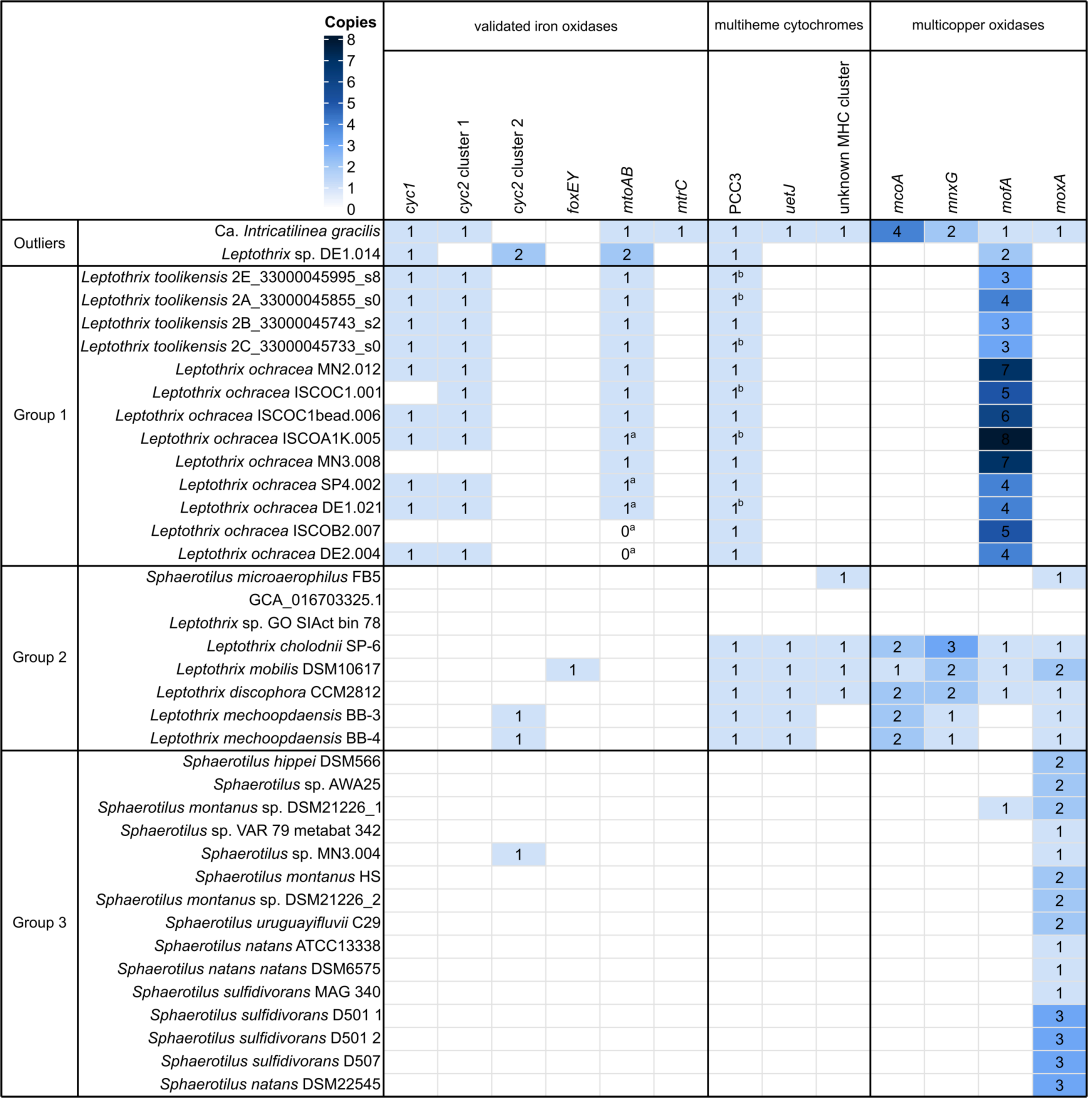
Metal oxidation genes encoded by SLG genomes. Shaded boxes indicate gene presence. Numbers indicate gene copy number. White boxes indicate gene absence. a = encodes additional truncated copies of *mtoA* and *mtoB*. b = encodes an additional extracellular cytochrome adjacent to a porin in the PCC3 gene cluster.

Genes for the functionally validated iron oxidase Cyc2 (55–58) were encoded in the majority of Group 1 genomes and in a limited number of genomes from Groups 2 and 3. Cluster 1 *cyc2* genes were predominantly associated with Group 1, and a single outlier (clusters defined by McAllister et al. [59]). Cluster 2 *cyc2* genes were identified in only three genomes from Groups 2 and 3, and one outlier genome (Fig. 5; Table S3).

Genes for a decaheme cytochrome-porin complex iron oxidase (*mtoAB*) (60, 61) are encoded in all Group 1 and outlier genomes (Fig. 5). The oxidative MtoA is a homolog to the iron-reducing MtrA; hence, the classification of these proteins was confirmed using a phylogeny of MtoA, MtrA, and PioA (Fig. S4). MtoA sequences from the SLG cluster with the functionally validated MtoA from *Sideroxydans lithotrophicus* ES-1; thus, their function is likely iron oxidation rather than iron reduction. These genes are absent from Group 2 and Group 3. “*Ca*. I. gracilis” is the only genome that contains the extracellular decaheme *mtrC,* and its *mtoA* gene is found in a singleton gene cluster, phylogenetically distinct from the other SLG *mtoA* genes (at the crown of Clade 2 in Fig. S4). A gene for the iron oxidase FoxE is encoded only in *L. mobilis* (Fig. 5).

**Fig 6:**
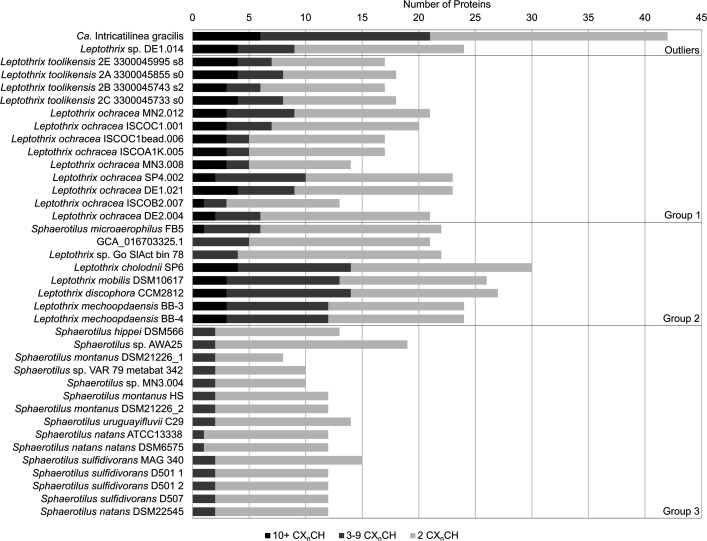
The number of genes encoding multiheme cytochromes. Proteins are categorized by the number of CXXCH, CXXXCH, and CXXXXCH heme-binding motifs.

Since the known iron oxidases are cytochromes, we searched the genomes for additional multiheme cytochromes, which could facilitate metal oxidation and extracellular electron uptake. All 38 genomes encode proteins with two or more heme-binding motifs (CX_2-4_CH; Fig. 6). Notably, Group 3 members encode very few proteins with more than two heme-binding motifs. In contrast, all members of Groups 1 and 2 encode at least four proteins with 3–9 heme-binding motifs, and the majority also encode proteins with 10 or more motifs. In summary, genomes from Groups 1 and 2 encode larger numbers of multiheme cytochromes, which also contain greater numbers of heme binding motifs per protein.

Porin cytochrome complexes (PCCs) are strong candidates for oxidizing various solid and dissolved Fe(II) substrates, transporting the electrons across the outer membrane. Iron-oxidizing bacteria frequently have functionally uncharacterized PCCs (62) that lack a demonstrated role in metal oxidation. We characterized each of the cytochromes with 10 or more heme motifs in Groups 1 and 2 with its name (if known), localization, and whether a porin was encoded within two genes on either side of the multiheme cytochrome gene (Table S4). Group 1 and one outlier encode MtoAB and a predicted porin-cytochrome complex known as PCC3 (Fig. 5) (62). Group 2 encodes PCC3 (cytochrome subunits containing 10–57 hemes, most commonly 21–29), the undecaheme UetJ (63), and its associated porin, and an unnamed decaheme cytochrome for which no nearby porin was identified. The second outlier genome, “*Ca*. I. gracilis” encodes one of each type: MtoAB, PCC3, UetJ, and the unnamed decaheme cytochrome. Overall, the analysis of multiheme cytochromes suggests that Groups 1 and 2 encode additional potential metal oxidases.

#### Multicopper oxidases

Multicopper oxidases (MCOs) serve diverse metabolic functions; some can catalyze either iron or manganese oxidation in bacteria (64–66). Nearly all SLG genomes encode at least one of the homologs to Mn-oxidizing MCOs encoded by *L. cholodnii* SP-6 (*mnxG*, *mcoA*, *moxA*, and *mofA* (67–70), but their distribution varies among groups (Fig. 5; Table S3). The *mcoA* and *mnxG* genes are found exclusively in Group 2 and the outlier “*Ca*. I. gracilis.” In contrast, *mofA* is characteristic of Group 1, in which multiple copies are common; *mofA* occurs rarely in Group 3 and is present in some Group 2 genomes as well as all outlier genomes. The *moxA* gene is present in all Group 3 genomes and one outlier. In Group 3, it is typically the sole MCO (and sole metal oxidase) encoded.

#### Sulfur

**Fig 7:**
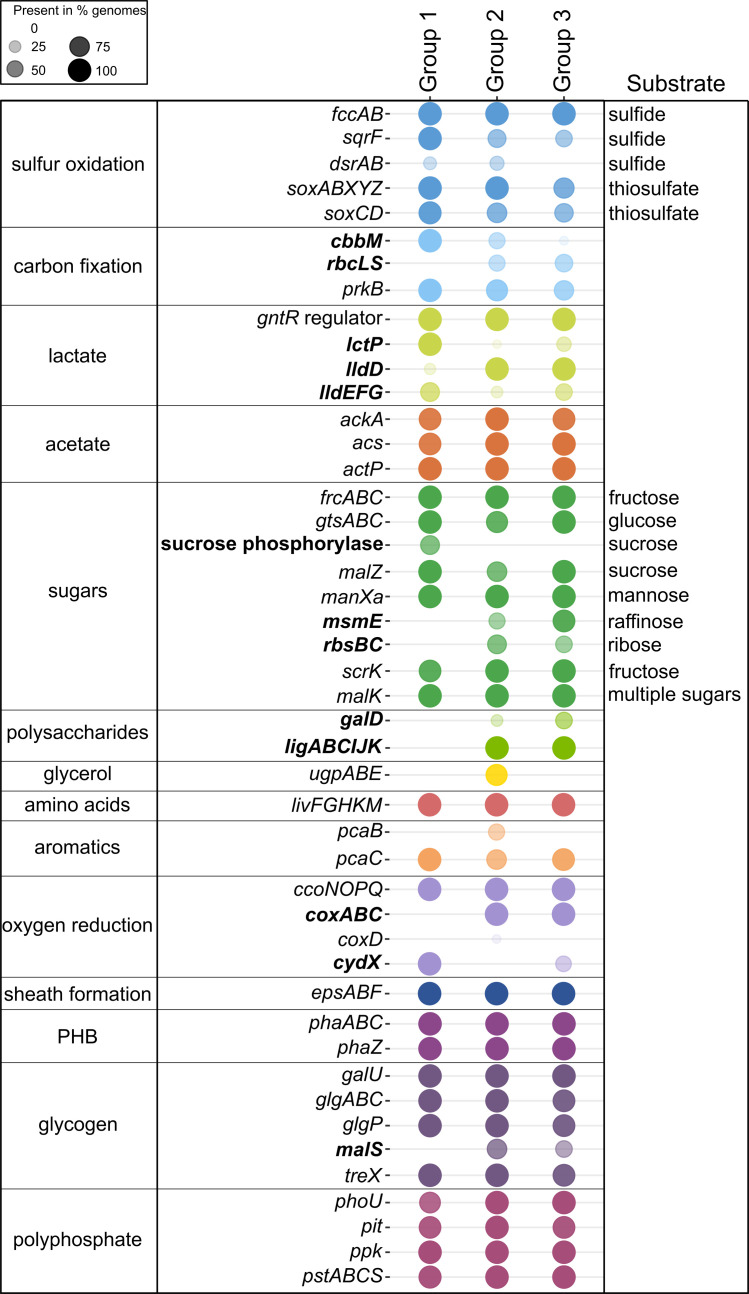
Genomic potential for metabolisms of interest. Based on the percentage of genomes in each SLG subgroup that encode each gene. Bolded genes are those with significant differences in presence between Group 1 and Groups 2/3.

The *sox* genes are involved in the oxidation of thiosulfate. The *soxABXYZ* genes encode a multi-enzyme complex involved in the initial steps of thiosulfate oxidation, while complete oxidation to sulfate typically requires *soxCD*. The *soxABX* genes are found in all members of Groups 1 and 2, and most members of Group 3, while *soxYZ* is ubiquitous across all genomes (Fig. 7; Table S3). The *soxCD* genes are found in most members of Groups 1 and 2. More than half of the Group 3 genomes encode *soxABXYZ* and *soxCD*, and the remaining six only encode *soxY* and *soxZ*. Overall, the results suggest that oxidation of thiosulfate is a viable energy-generating mechanism for the majority of SLG, although it is not a defining trait for Group 3 *Sphaerotilus*.

Sulfide dehydrogenase gene clusters (*fccAB*) are present in all 38 genomes in this study (Fig. 7). This gene is involved in dissimilatory oxidation of sulfide to elemental sulfur and represents a possible detoxification mechanism. A sulfide:quinone oxidoreductase gene (*sqrF*) was identified in all members of Group 1, and about half of the genomes from Groups 2 and 3.

The *dsrAB* genes were identified in roughly half of the genomes from Groups 1 and 2 (Fig. 7). The dissimilatory sulfite reductase DsrAB was originally shown to reduce sulfite to sulfide, although it has been shown to function in reverse to oxidize sulfide to sulfite (71, 72). In a maximum-likelihood protein tree, DsrA sequences from SLG cluster with the oxidative rDsrA sequences from S-oxidizing species (Fig. S5), indicating that the DsrAB in SLG is likely a reverse dissimilatory sulfite reductase. This suggests that some members of this group have a third mechanism for sulfur oxidation, but it is not a defining feature of SLG.

#### Carbon utilization

#### Carbon fixation

All genomes in this study, except four Group 3 *Sphaerotilus* members, have the genomic potential to perform carbon fixation. The key enzyme in the Calvin-Benson-Bassham (CBB) cycle for carbon fixation is RuBisCO, encoded by the genes *rbcL* and/or *rbcS* (Form I) or *cbbM* (Form II). RuBisCO Form II has a lower affinity for CO_2_ and accepts O_2_ more readily as an alternative substrate and competitive inhibitor compared to RuBisCO Form I; consequently, Form II is most effective under conditions where O_2_ is low and CO_2_ is relatively high, such as microaerobic environments (73). All genomes in Group 1, and four genomes in Group 2, encode Form II RuBisCO (cbbM), suggesting that these members preferentially grow in microaerobic environments. In contrast, genes for the small and large catalytic subunits of Form I RuBisCO (*rbcS/rbcL*) are found in the other four Group 2 genomes and nine Group 3 genomes (Fig. 7; Table S3). Form I is less susceptible to competitive inhibition by oxygen, and its presence in these organisms is consistent with growth under more oxic conditions. (For further discussion of oxygen preferences, see Supplemental text).

#### Organic carbon substrates

**Fig 8:**
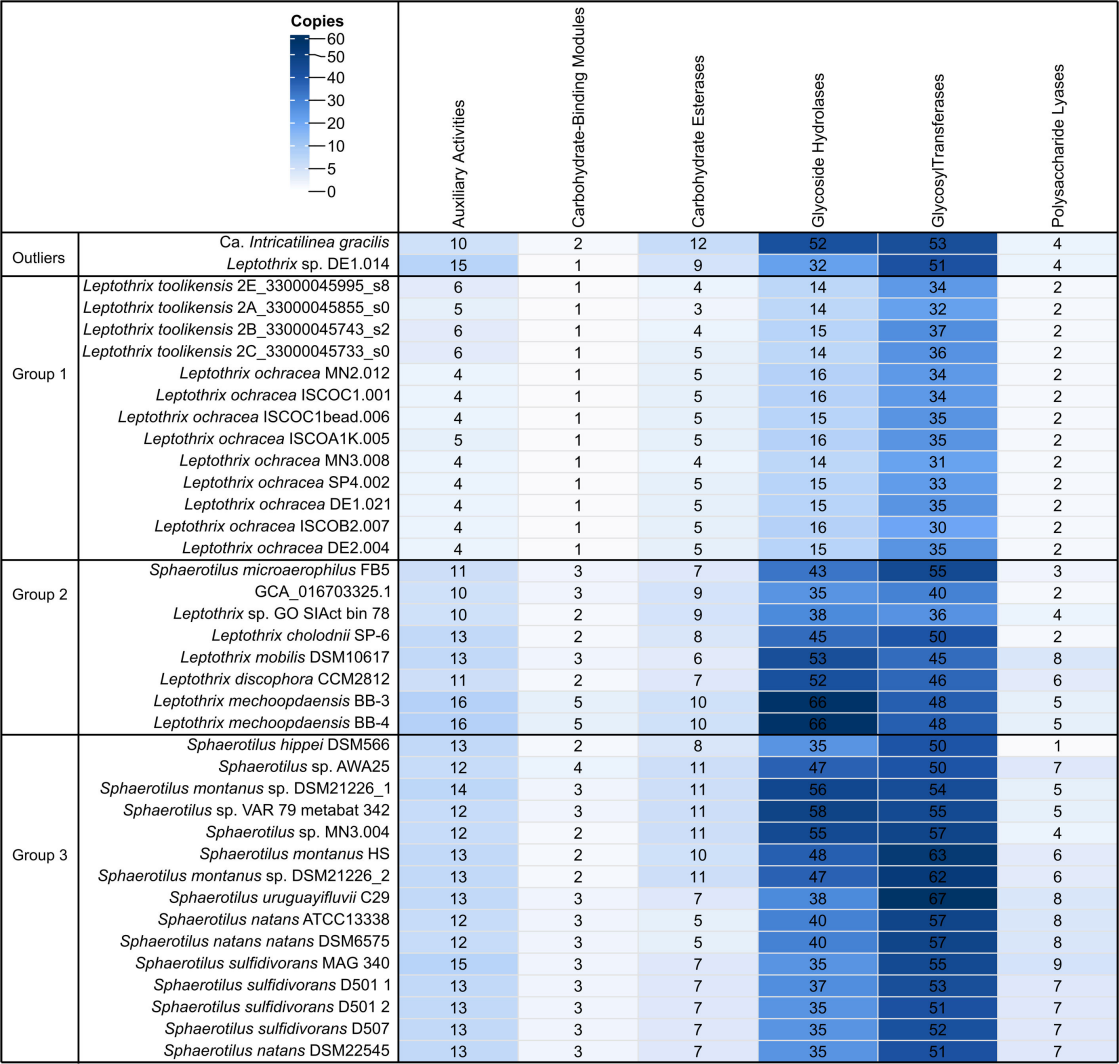
Potential for carbohydrate metabolism of SLG. Genes are summarized by category according to CAZyme annotations. Shaded boxes indicate gene presence. Numbers indicate gene copy number.

There are 602 gene clusters annotated as carbohydrate-active enzymes (CAZy), across six categories: auxiliary activities (AA), carbohydrate-binding modules (CBM), carbohydrate esterases (CE), glycoside hydrolases (GH), glycosyltransferases (GT), and polysaccharide lyases (PL) (Fig. 8). Group 1 genomes encode markedly fewer CAZy genes compared to Groups 2 and 3, with pronounced differences in the average numbers of glycoside hydrolase (GH) and glycosyltransferase (GT) genes. Specifically, Group 1 genomes contain an average of 15 GH and 34 GT genes, whereas Groups 2 and 3 encode averages of 45 and 43 GH genes, and 45 and 56 GT genes, respectively. These findings indicate a comparatively reduced genomic capacity for carbohydrate processing in *ochracea*-like *Leptothrix* relative to other members of the SLG.

Some genomes show potential for lactate utilization, while most genomes display acetate uptake capability. Lactate permease genes for lactate import (*lctP*) are encoded in all Group 1 genomes and in a minority of genomes from Groups 2 and 3 (Fig. 7; Table S3). Genes for metabolizing endogenous lactate (L-lactate dehydrogenase; either *lldD* or *lldEFG*) are found in all genomes, but their distribution varies: *lldD* is ubiquitous among Groups 2 and 3, and in the majority of *L. toolikensis* genomes, while *lldEFG* is ubiquitous among *L. ochracea* genomes and appears in less than half of genomes in Groups 2 and 3. Genes for a cation/acetate symporter (*actP*) are found in the full near-core gene set of the group (Fig. 7). All 38 genomes have at least one copy of this symporter, and the majority of genomes have multiple copies. Furthermore, all but two genomes have an acetate kinase (*ackA*) gene.

Genes for the import of glucose (*gtsABC*), fructose (*frcABC*), and mannose (*manXa*) are part of the near-core gene set of SLG. The glucose transport system *gtsABC* is present in 37 genomes, while *frcABC* and *manXa* are both ubiquitous (Fig. 7; Table S3). Fructokinase genes (*scrK*) are present in all but one genome. Furthermore, a gene for the ATP-binding subunit used by many organic carbon transporters (*malK*) is ubiquitous across SLG. These patterns indicate that nearly all members of SLG have the genomic potential to import and metabolize glucose, fructose, and mannose.

Genes for sucrose phosphorylase are found in all of the *L. ochracea* genomes and in one outlier genome (“*Ca*. I. gracilis”), while this gene is absent from *L. toolikensis* and Groups 2 and 3 (Fig. 7). This confers the potential to degrade sucrose into fructose and glucose-1-phosphate. Therefore, the potential for sucrose degradation appears to be primarily limited to *L. ochracea*.

Genomes from Groups 2 and 3 encode genes for ribose (*rbsBC*) and raffinose (*msmE*) transporters, which are absent from Group 1 (Fig. 7). The raffinose transporter *msmE* is part of the Group 3 near-core and occurs in some Group 2 genomes, while *rbsBC* is found in various members of Groups 2 and 3 but not in their near-core gene sets. These results show variability in sugar transporter presence within Groups 2 and 3 compared to Group 1.

Genes for a glycerol-3-phosphate transport system (*ugpABE*) are encoded by the majority of Group 2 genomes; however, they are absent from Groups 1 and 3 (Fig. 7; Table S3). Therefore, the potential for glycerol utilization is common among Group 2 genomes and unique to this group.

Genes encoding a branched-chain amino acid transport system (*livGFHMK*) are distributed across all SLG genomes (Fig. 7; Table S3). Each of the five *liv* genes is present in all 38 genomes, typically in multiple copies. These findings suggest that all members possess the genomic potential for branched-chain amino acid transport.

#### Sheath formation

The genes underlying sheath formation in SLG are not well understood and are difficult to separate from other metabolic functions, including EPS formation. However, polysaccharide export genes *wza*, *wzb*, and *wzc* could function in excreting polymers for sheath formation and are encoded by all genomes analyzed here (Fig. 7; Table S3). Additional genes related to organic carbon export and processing (e.g., glycosyl transferases) likely contribute to sheath development (74–77); however, additional work is needed to validate sheath formation genes in SLG.

## DISCUSSION

*Leptothrix* and *Sphaerotilus* have been known for over two centuries, beginning with *L. ochracea*, due to its conspicuous growth as rusty orange microbial mats and its clearly distinguishable sheaths tinted by iron oxides. *L. ochracea* lacks an isolate; hence, it no longer meets the standards for type material according to the International Code of Nomenclature of Prokaryotes (78; see also SeqCode [79]). However, extensive characterization of *L. ochracea* using xenic cultures, single-cell methods, and meta-omics has allowed for many of the same insights as would be available for an isolate, including its identity and physiology (20, 21, 33). In any case, according to the List of Prokaryotic names with Standing in Nomenclature (LPSN) (https://lpsn.dsmz.de/species/leptothrix-ochracea [80]), *Leptothrix* and *Leptothrix ochracea* are still the “correct names,” validly published by Kützing in 1843 (4) and included by Sneath et al. (81) in the Approved List of Bacterial Names.

The close phenotypic and genetic relationship between *Leptothrix* and *Sphaerotilus* has led to periodic renaming of species, causing confusion (6–8, 12, 18, 40, 44, 82). Recently, there have been renewed suggestions to rename all of the *Leptothrix* species to *Sphaerotilus* (26, 27); however, such a change requires careful consideration of all available evidence. Here, we perform a comprehensive assessment of the SLG by integrating analyses of phylogenomics, genome similarity, key functional genes, and phenotypes. Our analysis differs from previous ones in our inclusion of nine high-quality *L. ochracea* genomes plus four new genomes of the closely related *L. toolikensis*. With these additions, our results justify keeping the two genera, *Leptothrix* and *Sphaerotilus*, and add valuable insights into the distinguishing genetic features.

### Classification

Existing genome classification tools have previously been inconsistent in classifying SLG genomes, partly due to few reference genomes in databases and also the lack of a systematic phylogenetic analysis using the full SLG diversity. Our work provides a comprehensive data set with multiple representatives from each of the three subgroups and helps establish a framework for more rigorous genomic classification. This framework will allow recognition of more genomes as SLG from existing or new data sets, which will add further resolution for improving classifications. Any current and future classifications of new genomes should be supported by careful application of the polyphasic approach outlined below, using concatenated conserved (e.g., ribosomal) protein trees, genome comparison metrics, environmental ecotypes, and consideration of genotypic and phenotypic lines of evidence.

When using genomes to help delineate genera, the recommendation is to use multiple genome comparison metrics alongside phylogenomics and analyses of genes that represent shared physiology and ecological niche, since thresholds for quantitative metrics like ANI or AAI are not definitive (52). These latter metrics have been used to support the merging of *Leptothrix* and *Sphaerotilus* into a single genus; Smolyakov et al. (26) applied a 65% AAI cutoff, while Liu et al. (27) used an AAI threshold of 67.12%–71.55%, specific to the Sphaerotilaceae. When developing the combined ANI/AF genome relatedness index, Barco et al. (54) found that family-specific thresholds are required due to differences in evolutionary rates between taxa. Boundary-defining thresholds often need to be adjusted to ensure coherence with phylogenomics (52, 83, 84). Therefore, as in previous SLG classification, using ANI or AAI values to delineate genera is still only an approximate estimation that needs genotypic, phenotypic, and environmental context to provide a complete taxonomic picture.

The phylogenetic analyses presented here support the existence of three subgroups within *Sphaerotilus-Leptothrix*: the *ochracea*-type *Leptothrix* (Group 1), the *mobilis*-type *Leptothrix* (Group 2), and *Sphaerotilus* (Group 3). Here, AAI results suggest that while Groups 2 and 3 could be considered the same genus, Group 1 belongs to a separate genus. Using the ANI/AF thresholds for the families that SLG have belonged to previously (Burkholderiaceae, Comamonadaceae), the genus boundary lies between Group 1 and Groups 2/3. This is supported by the phylogenetic analysis, in which the Group 2 *Leptothrix* and Group 3 *Sphaerotilus* cluster more closely with one another than either group does with Group 1, suggesting that the genus *Leptothrix* may be paraphyletic. Genome size and GC content provide further evidence of Group 1’s distinctiveness. The genomes are ~60% the size of Group 2/3 genomes, indicating that Group 1 genomes have been streamlined. Group 1 has an average %GC that is consistently 8% lower than Groups 2/3, giving further evidence of a diverging evolutionary trajectory. Given this combination of phylogenomics and multiple genomic comparison metrics, Group 1 clearly stands apart.

The current standards for defining genera require a polyphasic approach that considers phylogeny, phenotypes, and environmental niches, not just genomic metrics (29, 41, 54, 85–93). Looking at the distribution of key functional genes, genotype patterns mirror phenotypes, supporting the above findings for Group 1 but causing some difficulty in characterizing Group 2. Group 1 is unique in that all members encode iron oxidases (Cyc2, MtoAB) that can yield energy, and multiple copies of *mofA*, consistent with findings that *L. ochracea* requires Fe(II) for growth (33). Group 3 almost all lack genes for iron oxidation energy metabolism, and generally only encode one gene for metal oxidation, the MCO *moxA*, suggesting that metal oxidation is not central to Group 3 energy metabolism. In contrast to Groups 1 and 3, metal oxidation gene patterns in Group 2 are less consistent, making it difficult to determine defining features. Perhaps the most notable feature of Group 2 is that many genomes have multiple metal oxidation genes, although most lack the well-characterized genes for iron oxidation, making it unclear whether Group 2 members generally oxidize iron for energy. Group 1 genomes host relatively few organic carbon utilization genes, which fits with isotopic and transcriptomic evidence of mixotrophy in *L. ochracea* (21, 33). This contrasts with Groups 2 and 3, in which all isolates grow organoheterotrophically; this is reflected in the abundance and range of organic utilization genes (e.g., CAZy) in Groups 2 and 3 genomes. Among Groups 2 and 3, the distribution of genes for metal oxidation and abundance of genes for organic carbon utilization fit with the fact that neither requires substantial quantities of iron for energy, instead relying on organotrophy for energy metabolism.

Group 2 members lack homogeneity in genotype, phenotype, and ecotype, which makes it difficult to determine if Group 2 (or perhaps a subset) should be reassigned to *Sphaerotilus*. There is some evidence that Group 2 may not be well-defined and instead includes two subgroups that display phylogenetic separation consistent with their isolation sources. The majority of the group is formed by five freshwater *Leptothrix* isolates, all of which are metal oxidizers with multiple potential metal oxidase genes. In contrast, the three genomes derived from sludge (including one isolate) largely lack metal oxidases and instead appear to be organotrophic (45). Consequently, the freshwater Group 2 isolates differ markedly from *Sphaerotilus*, whereas the three Group 2 members derived from sludge do not. These three are in the minority, and they have a markedly lower alignment fraction to *L. mobilis* than the metal-oxidizing members of Group 2 (Fig. 3; Table S2) and form a phylogenetically distinct clade, suggesting that they could be considered as distant from *L. mobilis* as any Group 3 genome. The bootstrap of the node defining Group 2 is 63%, and it is possible that the addition of more genomes will cause this organotrophic subgroup to associate with Group 3 instead of Group 2. In order to make a definitive decision, additional isolates related to both subgroups are needed to more confidently define Group 2.

We predict that further genomic sequencing and phenotypic characterization of isolates will result in well-supported (i.e., high bootstrap value, >75%), coherent, phylogenomic, and phenotypic/genotypic groups. For now, Group 1 (*L. ochracea* and *L. toolikensis*) shows sufficient phylogenetic and functional uniqueness to merit a genus distinct from *Sphaerotilus*. Overall, genus delineation should consider combined genotype and phenotype, alongside phylogenomics and genomic metrics. This polyphasic framework gives us multiple coherent lines of evidence for retaining the *Leptothrix* designation for *L. ochracea* and *L. toolikensis,* while further isolates and genomes are required to conclusively classify Group 2; therefore, the current genus designations should be maintained until this is resolved.

### Interpreting genes in the context of classic phenotypic distinctions

There are three canonical phenotypic distinctions, which have been used to identify SLG genera and species: iron and manganese oxidation, response to organics, and sheath morphology. Here, we consider how this SLG pangenome analysis gives insight into each of these phenotypic categories.

#### Iron and manganese oxidation

Historically, *Leptothrix* and *Sphaerotilus* were distinguished by their metal oxidation ability. While both are able to oxidize iron, only *Leptothrix* can oxidize manganese (10, 94). The genomes show a multitude of known and putative metal oxidation genes that reveal more about metal oxidation potential. However, the genotypes do not all cohere to the phenotypic distinctions, but given the ongoing research on metalloxidases, this suggests that SLG genomes include novel iron oxidases.

Known microbial manganese oxidases are multicopper oxidases, including the four encoded in the SLG pangenome: McoA, MnxG, MofA, and MoxA (67–70). While other MCOs are known to oxidize iron (65, 95, 96), these four are not known to play roles in iron oxidation. Almost all of the SLG genomes encode at least one of these four MCOs, but if they are all truly manganese oxidases, this conflicts with the understanding that *Sphaerotilus* do not oxidize manganese. *Sphaerotilus* do oxidize iron, but all but one Group 3 *Sphaerotilus* lack known or suspected iron oxidation genes, leaving the MCO MoxA homolog as the only metalloxidase, suggesting that it is a plausible iron oxidase, although this has yet to be tested.

The currently known iron oxidases are Cyc2, MtoAB, and FoxEY, while PCC3 and UetJ are predicted porin cytochrome clusters present in other iron-oxidizing bacteria and could theoretically oxidize various Fe(II) substrates, including dissolved free Fe^2+^, organic-chelated Fe^2+^, and mineral-bound Fe^2+^, which vary in redox potential. Group 1 and most of the Group 2 genomes contain these known and/or putative iron oxidases, consistent with the iron-oxidizing phenotype of *Leptothrix*. The presence of multiple iron oxidases is common among iron-oxidizing bacteria (97), and different iron oxidases may be expressed to gain energy from distinct iron substrates (61, 98).

#### Carbon utilization: autotrophy, mixotrophy, and heterotrophy

Another classical distinction in SLG is “response to organics.” A stronger response in culture suggests organoheterotrophy, while a muted or non-response implies mixotrophy, oligotrophy, or autotrophy. Most SLG genomes possess genes for both carbon fixation and heterotrophy; so, in theory, SLG members could be autotrophic, mixotrophic, and heterotrophic, but it is an open question as to how much each group or species can exhibit autotrophic vs. heterotrophic character. There has been relatively little systematic exploration of this, as evidence for autotrophy/mixotrophy is sparse. The only isotope-labeled carbon uptake experiments have been performed in xenic cultures of *L. ochracea*, which show some uptake of inorganic carbon in the presence of organic carbon (33). This demonstrates mixotrophy, consistent with poor responses to organics in *L. ochracea* cultures, the lower number of heterotrophy genes, and metabolic modeling results (10, 21, 37). The only clear experimental evidence of autotrophy is for the isolate *S. natans* D-507, which was shown to grow autotrophically by sulfur oxidation (25). Most SLG genomes possess both sulfur oxidation and carbon fixation genes, and, thus, the potential for lithoautotrophic sulfur oxidation, but this is untested in other SLG, as is lithotrophic iron oxidation.

Mixotrophic iron oxidation is most plausible in SLG Group 1 but is also tenable in some of Group 2, while demonstration of autotrophic iron oxidation remains elusive. Iron oxidation yields relatively little energy and also requires reverse electron transport, making autotrophic iron oxidation a challenging lifestyle (36). The presence of various organic transporters and utilization genes, even in Group 1 genomes, suggests that SLG have adapted to use organics where available, tending toward mixotrophy and organoheterotrophy. The variability in organic utilization genes, particularly in Groups 2 and 3, where they occur in higher numbers, is consistent with the variation in substrates that can support SLG growth (10). Thus, the overall diversity in carbon metabolism likely accounts for the ability of SLG to colonize a range of aquatic environments, but it remains to be seen the range and flexibility of each SLG in terms of organic and inorganic carbon utilization.

#### How metal and carbon metabolisms influence sheath morphology

Before the advent of molecular taxonomy, sheath morphology played an important role in characterizing SLG. The starkest difference lies in the comparison of *L. ochracea* sheaths, which are mainly empty, versus Groups 2 and 3 sheaths that are filled with cells. These differences in sheath habits can be explained, given our understanding of SLG metal oxidation and carbon metabolism geno/phenotypes.

The typical SLG sheaths of the Groups 2 and 3 isolates are organic. Studies of *L. cholodnii* showed that terminal cells at either end of a filamentous chain excrete sheath fibrils that form a robust organic matrix held together by disulfide bonding (99); the *S. natans* sheath is also proposed to form similarly (100). The organic backbone of these sheaths is consistent with an organoheterotrophic metabolism, and metal oxidation only occurs once sheaths are formed (101, 102). Because the organotrophic SLG do not require metal oxidation for growth, less metal is oxidized per cell, and the sheaths are not completely encrusted; so, the cells can continue to inhabit sheaths without being cut off from nutrient supply. The resulting morphology is long filaments of cells encased in a manganese and/or iron oxyhydroxide-coated sheath.

In contrast, Group 1 appears to exhibit more autotrophic character in phenotype and genotype. Group 1 has multiple pathways to connect iron oxidation to electron transport and energy generation (Cyc2, MtoAB, and PCC3) but relatively few organic utilization genes. This supports the likely niche of Group 1 *Leptothrix* as mixotrophs that use iron oxidation as a major energy-generating mechanism, which would result in copious oxides. The largely empty sheaths of *L. ochracea* could be considered analogous to the stalks of the autotrophic iron oxidizer *Gallionella*, in which one end is anchored as a holdfast, and cells package ferric iron waste in a mineralized stalk with a cell at the leading end (31). Vesenka et al. (74) showed that *L. ochracea* sheath formation is driven by filaments of 10–20 cells moving in aqueous media and simultaneously excreting nanofibrils that are immediately encrusted with (and largely composed of) ferric oxyhydroxide byproducts of their metabolism. In essence, as cells form the sheaths and move past iron oxides, they are performing a unique form of metal-mediated motility. The result is a trailing, empty tubular mineralized sheath that is the signature of *L. ochracea*, again reflecting its mixotrophic iron oxidation metabolism.

### Conclusions and implications

This work provides a comprehensive view of the SLG, giving a robust framework for consistent identification and interpretation. The SLG is separated into three functional subgroups based on a polyphasic classification that incorporates phylogenomic and functional evidence. Combining Groups 2 and 3 as *Sphaerotilus* is justifiable based on phylogeny, genome similarity, and, to some extent, functional potential. However, there are clear distinctions between Group 2 and Group 3 in metal oxidation genes, which merit distinguishing these subgroups; the diversity in Group 2 relative to the number of genomes suggests that more genomes/isolates are needed to resolve its relationship to the other groups. For now, we recommend retaining the existing genus designations for Group 2 until further genomes improve the phylogenetic resolution. The clear differences between Group 1 and Groups 2/3 require the retention of the genus *Leptothrix* for members of Group 1, rather than fully combining the two genera as one (8, 26, 27, 40).

Genus nomenclature is important for understanding and communicating how microbes influence environmental outcomes. The metabolic differences between the more autotrophic/mixotrophic vs. organoheterotrophic SLG result in distinct biogeochemical and ecological roles. The organoheterotrophic *Leptothrix* and *Sphaerotilus* likely oxidize much less iron and thus have more influence on carbon rather than metal cycles and restrict these taxa to more organic-rich environments. In contrast, Group 1 oxidizes large quantities of iron in the environment, thereby acting as major drivers of iron cycling and resulting nutrient and metal sequestration and release. Their ability to grow in low carbon environments and produce iron-mineralized sheaths makes them keystone species that engineer mat structures which are colonized by diverse microbial communities. These iron mats are critical biogeochemical hotspots that occur where groundwater discharges into surface water, and being able to precisely identify and interpret metabolisms of Group 1 *L. ochracea* and *L. toolikensis* will improve our understanding of freshwater metal and nutrient cycles.

### Description of *Leptothrix toolikensis* sp. nov

*Leptothrix toolikensis* (too.li.ken’sis. N.L. fem. adj. of Toolik, referring to the Toolik Lake Field Station; from the Iñupiaq word tutlik, meaning yellow-billed loon). The organism was identified from four metagenome-assembled genomes recovered from an iron seep adjacent to the Oksruykik River, North Slope, AK, USA. Genomes assigned to this species range from 2.67 to 2.87 Mbp in length with a G+C content of 60.9%–61.2%. Phylogenomic analysis places the organism within the genus *Leptothrix* in the family Sphaerotilaceae. Its closest relative is *L. ochracea*. A 16S rRNA gene sequence exists for the genome *L. toolikensis* 2E 3300045995 s8. Genomic analysis revealed the potential for lithotrophic iron oxidation, carbon fixation via the CBB cycle, and utilization of organic matter, indicative of a mixotrophic metabolism for cell growth. The type material is the genome sequence *L. toolikensis* 2C 3300045733 s0.

### Description of *Leptothrix mechoopdaensis* sp. nov

*Leptothrix mechoopdaensis* (me.choop.da.en′sis. N.L. fem. adj. referring to Mechoopda, meaning “the marshy place,” and honoring the Mechoopda tribe whose ancestral lands encompass the isolates’ original iron seep). Cells grow as streptobacilli within a sheath, as observed from cultures grown under laboratory conditions. Cells grow on Lepto medium plates with MnCO_3_ and FeSO_4_ (16, 48). Strains BB-3 and BB-4 were isolated from an iron seep near Centerville, CA, USA. Genomes assigned to this species are 5.07 Mbp in length with a G+C content of 69.7%. Phylogenomic analysis places the organism within the *Sphaerotilus-Leptothrix* group in the family Sphaerotilaceae. Its closest relative is *L. discophora*. A 16S rRNA gene sequence exists for both isolates. The type material is the isolate strain *L. mechoopdaensis* BB-3.

## MATERIALS AND METHODS

### Genome collection

Thirty-eight high-quality genomes were included in this analysis (Table S1). Eleven MAGs were reconstructed from iron microbial mats at the Savannah River Site and from Spruce Point as described in Tothero et al. (21). Two *L. mechoopdaensis* isolates were sequenced from an iron seep in Centerville, CA, in Butte Creek Canyon as described in Fleming et al. (48).

Four MAGs (*L. toolikensis*) were reconstructed from samples collected at a unique iron seep adjacent to the Oksruykik River (68.6722, −149.1352), near the Toolik Lake field station (North Slope, AK) (47). Four separate samples, each consisting of 50 mL of aqueous mat, were collected on June 28, 2019, for metagenome analysis and MAG reconstruction. Each sample was concentrated by centrifugation and resuspended in 1 mL of deionized water. This suspension was added to a Powersoil Pro (Qiagen) extraction tube for DNA isolation. DNA was sent to the Joint Genome Institute for sequencing and metagenomic analysis. Illumina libraries were constructed and sequenced using the Illumina NovaSeq S4 platform, generating 2 × 151 paired-end reads; 132,294,094 reads totaling 19,976,408,194 bp were generated.

BBDuk v38.90 was used to remove contaminants and trim adapter sequences, homopolymers, and regions where the quality score drops to 0 (103), as well as to remove reads that contained four or more “N” bases, had an average quality score less than 3, or had a minimum length ≤51 bp. Reads were mapped with BBMap and filtered to remove reads that mapped to common eukaryotic (humans, cats, dogs, and mice) and microbial contaminants. Reads were corrected using bbcms v38.86 (103). The readset was assembled using metaSPAdes v3.14.1 using kmer lengths of 33, 55, 77, 99, and 127 (104). The input read set was mapped to the final assembly, and coverage information was generated with BBMap v38.86 (103).

The IMG/M, NCBI Genome, GenBank, and RefSeq databases were searched for existing genomes classified as *Leptothrix* and *Sphaerotilus*. In addition, the existing IMG BLAST all database was used to BLAST three ribosomal proteins (S8, L6, and L4; selected based on those identified in Olm et al. (105) for species-level delineation) from *L. cholodnii* and *L. mobilis*. Genomes with a hit >70% identity to at least one of these ribosomal proteins were pulled (*n* = 1,327), and hits were screened for genomes not classified to the genus level (*n* = 51). Genome fasta files were downloaded from IMG, and genomes were classified as *Leptothrix* and *Sphaerotilus* based on concatenated ribosomal protein trees.

All 16S rRNA gene sequences (*n* = 863,832) from GTDB genomes passing QC (≥200 bp, E-value ≥ 1e−06) were downloaded from the GTDB-tk release r220. Sequences were filtered to include only those in the Burkholderiaceae family (*n* = 21,725), and these were placed in a maximum-likelihood tree. The smallest monophyletic clade containing all verified *Leptothrix* and *Sphaerotilus* was selected from this tree, and full genomes from unclassified sequences within that clade were downloaded from GenBank and RefSeq for further classification.

### Tree generation

Alignments were generated using MUSCLE v.5.1 (106) and trimmed and masked (sites with >30% gaps) in Geneious Prime. Maximum likelihood trees were generated in RAxML v1.2.1 with 1,000 bootstraps, using the LG+G model for trees of amino acid sequences and GTR+G model for trees of nucleotide sequences (107, 108). Trees were visualized in iTOL v7 (109).

### Genome classification and data set curation

Genomes were annotated in Anvio-v8 (110) using Prodigal v.2.6.3 (111). Concatenated ribosomal protein trees were generated using alignments of 39 large and small ribosomal protein sequences (S12, L1, L13, L14, L16, L17, L18p, L19, L2, L20, L21p, L22, L23, L27, L27A, L28, L29, L3, L32p, L35p, L4, L5, L6, L9_C, S10, S11, S13, S15, S16, S17, S19, S2, S20p, S3_C, S6, S7, S8, S9, and L24). A phylogenetic tree was generated using the Up-to-date Bacterial Core Genes 2 pipeline using 81 core bacterial genes (112, 113), using prodigal v2.6.3 for gene calling (111), MAFFT v7.525 for alignment (114), and RAxML v8.2.13 for tree construction (107). AAI was calculated using EzAAI v.1.2.4 (115). Genome quality was determined using CheckM v1.0.18 (116). Average Nucleotide Identity (ANI) and Alignment Fraction (AF) were calculated using FastANI v0.1.3 (117, 118) and the ANIcalculator 2014-127, version 1.0 (https://ani.jgi.doe.gov/html/download.php) (119). Percentage of conserved proteins (POCP) was calculated using the Bio-py POCP-matrix calculator (53, 120).

Genomes were included in the analysis based on (i) clustering with a known *Leptothrix* or *Sphaerotilus* genome in the concatenated tree; (ii) >70% AAI to at least one known *Leptothrix* or *Sphaerotilus* genome; and (iii) greater than 90% completeness and less than 5% contamination. The final filtered data set, referred to as “SLG” contained 38 genomes. Genome groups, referred to as the “*ochracea*-type *Leptothrix*” or Group 1 (*n* = 13), “*mobilis*-type *Leptothrix*” or Group 2 (*n* = 8), “*Sphaerotilus*” or Group 3 (*n* = 15), or “outliers” (*n* = 2), were separated based on monophyletic clades in the concatenated ribosomal protein tree.

### Pangenome generation

Anvio-v8 was used to generate a pangenome database of the final filtered data set (110, 121). Genes were clustered using a min-bit parameter of 0.5 and an mcl inflation parameter of 2, and using NCBI blastp for sequence similarity (122–124) and hierarchical clustering. Functional annotations were assigned to gene clusters in using FeGenie (125), NCBI COGs, Pfams, Kegg Kofams, CAZymes, a modified heme counter script to search for CXXCH, CXXXCH, and CXXXXCH motifs (126), and a custom Python script that detected conserved MCO motifs. Near-core gene sets were identified based on the presence of gene clusters in > 85% of genomes in the final filtered data set or within each genome group. Venn diagram generation was done using goodcalculators.com/venn-diagram-maker/.

### Gene sequence analyses

The localization of protein sequences with 10 or more heme-binding motifs was determined using PSORTb v3.0.3 (127). If needed, signal peptides were identified with SignalP 6.0 and/or Phobius (128–130). Porins were searched for using a combination of protein localization prediction, BLAST, and HHpred (131, 132).

To predict the oxidative vs. reductive function of DsrA in SLG, a maximum-likelihood protein tree was constructed using reference sequences from Loy et al. (72). To validate the FeGenie annotations of MtoA sequences, a maximum-likelihood protein tree was constructed using reference sequences (133), including functionally validated MtoA, MtrA, and PioA reference sequences.

Data visualization was performed in R v4.3.2 using the tidyverse ecosystem. Scatter plots and bubble plots were generated using ggplot2 (134). Heatmaps were visualized using pheatmap and complexheatmap (135, 136). Dendrograms were generated using dendextend (137).

## Data Availability

Genome sequences for *Leptothrix mechoopdaensis* BB-3 and BB-4 are deposited in NCBI under BioProject PRJNA1336152, under GenBank accessions GCA_053893855.1 (BB-3) and GCA_053893815.1 (BB-4). Genome sequences for *Leptothrix toolikensis* strains are deposited in NCBI under BioProject PRJNA1438219. The taxonomic names proposed in this study are established under SeqCode at https://registry.seqco.de/registers/r:3pq_uc4e.

## SUPPLEMENTAL MATERIAL

Supplemental figures and text msystems.01768-25-s0001.pdf Supplemental text on genomes and Figures S1-S5.

Supplemental Tables msystems.01768-25-s0002.xlsx Tables S1-S4.

ASM does not own the copyrights to Supplemental Material that may be linked to, or accessed through, an article. The authors have granted ASM a non-exclusive, world-wide license to publish the Supplemental Material files. Please contact the corresponding author directly for reuse.
