# Supplementary material for: Distinguishing Leptothrix and Sphaerotilus genera by an integrated genomic-phenotypic analysis supported by new Leptothrix genomes
Source: mSystems. 2026 Jun 12;11(7):e01768-25. doi: 10.1128/msystems.01768-25 (PMC13386937; doi:10.1128/msystems.01768-25)
Supplement: Supplemental figures and text — Supplemental text on genomes and Figures S1-S5. [file msystems.01768-25-s0001.pdf]

Supplemental information for Distinguishing *Leptothrix* and *Sphaerotilus* genera by an integrated genomic-phenotypic analysis supported by new *Leptothrix* genomes

Authors: Gracee K. Tothero, Jessica L. Keffer, David Emerson, Emily J. Fleming, Clara S. Chan

Table of Contents

Supplemental Results Text

Supplemental Figures

- Figure S1. Concatenated protein tree of the *Sphaerotilus-Leptothrix* group based on 81 bacterial core genes.
- Figure S2. Heatmap of percentage of conserved proteins between SLG genomes.
- Figure S3. Average nucleotide identity (ANI) versus alignment fraction (AF) plot by FastANI
- Figure S4. Maximum likelihood tree of MtoA, MtrA, and PioA sequences.
- Figure S5. Maximum likelihood tree of DsrA sequences.

Supplemental References

The following supplemental tables can be found in the associated excel file:

- Table S1. Genome attributes
- Table S2. Average Nucleotide Identity (ANI) and Alignment Fraction (AF) values
- Table S3. Gene annotations
- Table S4. Summary of predicted multiheme cytochrome gene clusters

Supplemental Results Text

All genomes used in this study have been previously published ((1–17); **Table S1**), with the exception of four *L. toolikensis* genomes, which were reconstructed in this study.

Oxygen Reduction

Genes encoding the *cbb3*-type cytochrome *c* oxidase (*ccoNOPQ*) are encoded by all SLG genomes (**Figure 7; Table S3**). This is consistent with growth under microoxic conditions, as this terminal oxidase has a high affinity for oxygen (18). Genes encoding the *aa3*-type cytochrome *c* oxidases (*coxABC*) are encoded by all members of Groups 2 and 3, yet are absent from Group 1. This terminal oxidase is consistent with growth under more oxic conditions, as this terminal oxidase has a low affinity for oxygen (19–21). The gene patterns observed in both terminal oxidases and RuBisCO genes (below) reflect broader oxygen tolerance among Groups 2 and 3 compared to a preference for microaerobic growth in Group 1.

Vitamin B12 transport

A vitamin B12 transporter gene (*btuB*) is encoded in all genomes within Group 1 and Group 3 (**Figure 7; Table S3**). However, it is only present in four Group 2 genomes. This transporter is dependent on energy coupling to TonB; the *tonB* gene is encoded in all 38 genomes, but has multiple functions beyond B12 transport.

Organic Carbon Storage Polymers

Members of SLG are commonly observed to accumulate polyhydroxybutyrate (PHB) granules (22). All four of the genes for synthesis (*phaABC*) and depolymerization (*phaZ*) are ubiquitous across SLG (**Figure 7; Table S3**). Glycogen synthesis and polymerization is also common; the *glgABC* genes are present in all genomes (**Figure 7**). Genes for glycogen depolymerization include *galU*, *glgP*, *glgY*, *malS*, and *treX*. Nearly all genomes have *galU* and *glgP*, but none have *glgY* (**Figure 7**). MalS, which degrades glycogen and maltodextrin, is found in some Group 2 and Group 3 genomes, but is absent from Group 1. In sum, these results show the use of intracellular polymers for organic carbon storage is a common trait of SLG, and polysaccharide storage is not limited to *Sphaerotilus*, as has been previously reported (23).

Polyphosphates

Genes with a role in polyphosphate accumulation include *pit*, *pstABCS*, *phoU*, and *ppk*. A phosphate ABC transport system (*pstABCS*) is present in all 38 genomes, as is the polyphosphate kinase (*ppk*) gene, while the phosphate transporter genes *phoU* and *pit* are each absent from two genomes (**Figure 7; Table S3**). Consequently, the ability to accumulate polyphosphates is near-ubiquitous across SLG.

Supplemental Figures

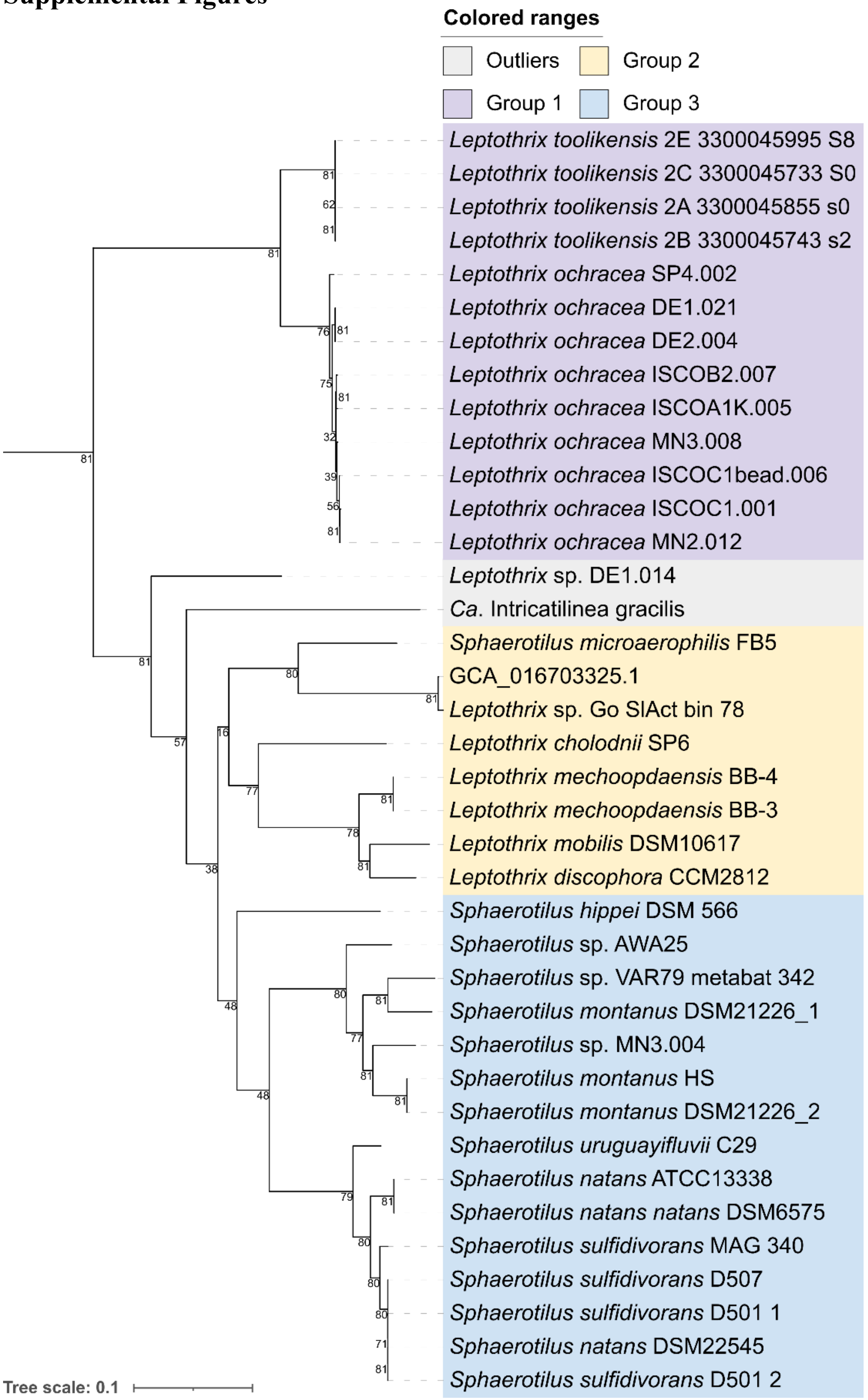

Figure S1. Concatenated protein tree of the *Sphaerotilus-Leptothrix* group based on 81 bacterial core genes. Constructed using the UBCG2 pipeline using prodigal v2.6.3 for gene calling, MAFFT v7.525 for alignment, and RaxML v8.2.13 for tree construction.

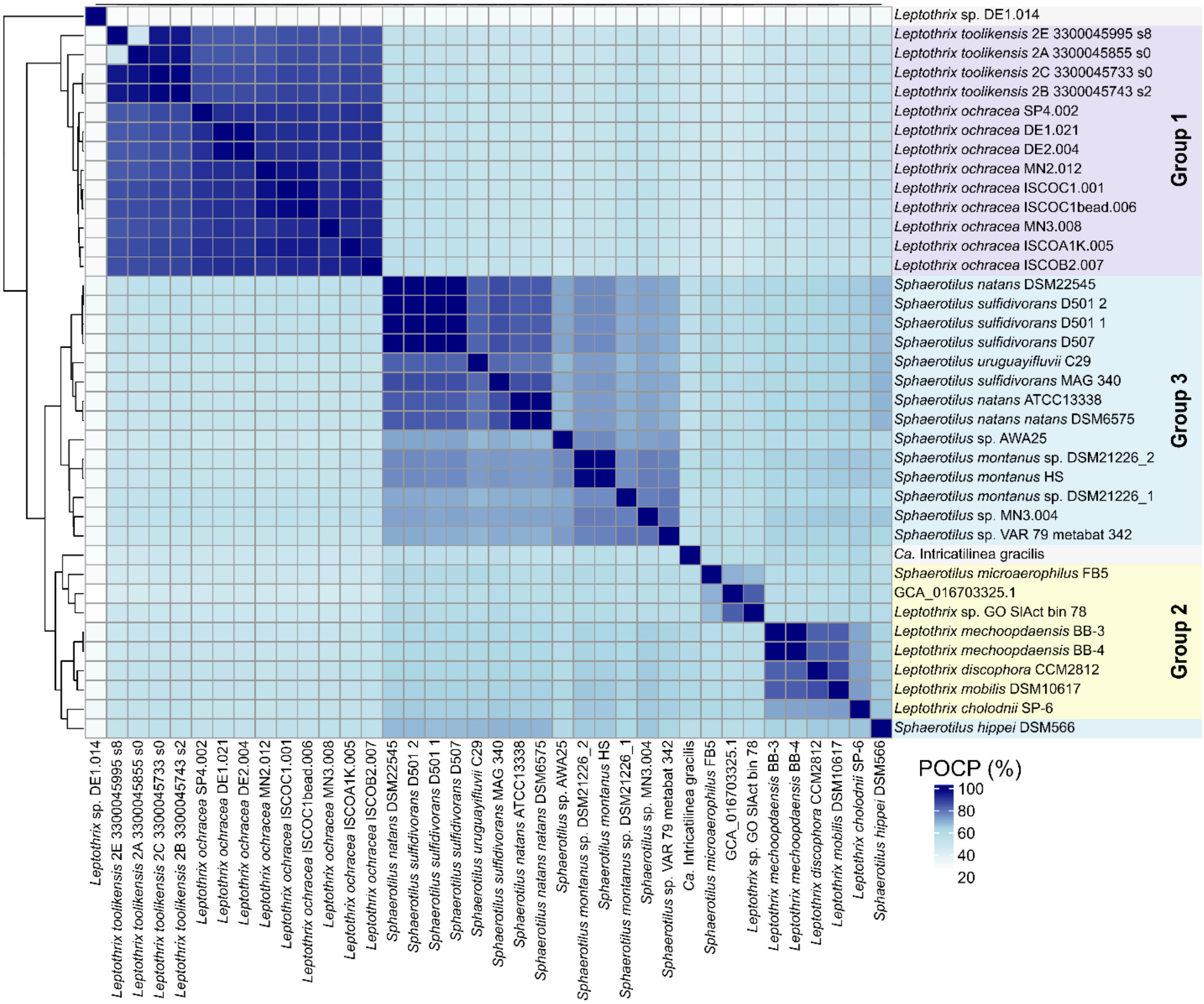

Figure S2. Heatmap of percentage of conserved proteins between SLG genomes. Calculated using the Bio-py POCP-matrix calculator.

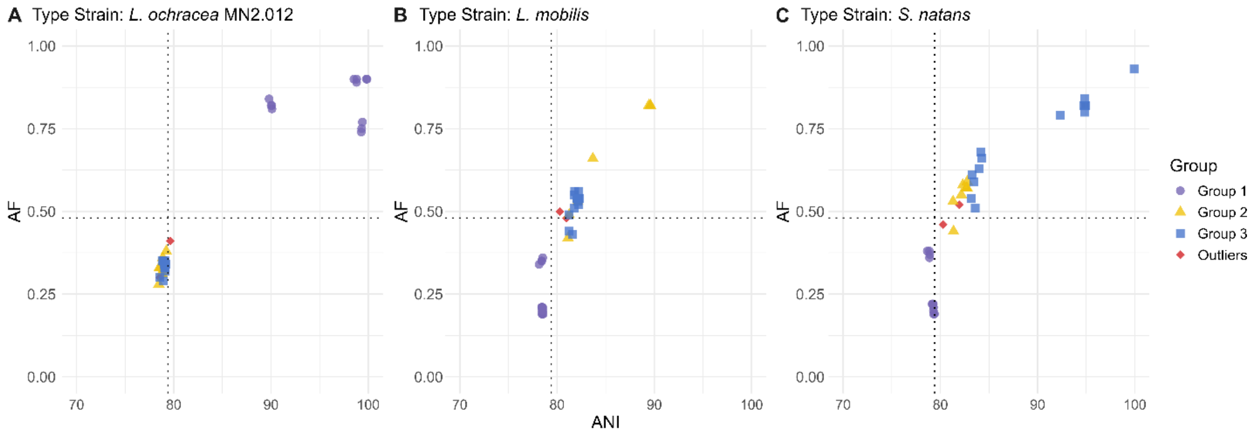

Figure S3. Average nucleotide identity (ANI) versus alignment fraction (AF) for all genomes in this study, compared to each of three type strains: (A) *Leptothrix ochracea* MN2.012, (B) *Leptothrix mobilis* DSM10617; (C) *Sphaerotilus natans* CCM2812. Each panel shows pairwise comparisons to the type strain. Dashed lines indicate genus-level thresholds of 79.40% ANI and 0.480 AF, averages of the thresholds for Burkholderiaceae and Comamonadaceae defined by Barco et al. (24). Calculated using FastANI v0.1.3.

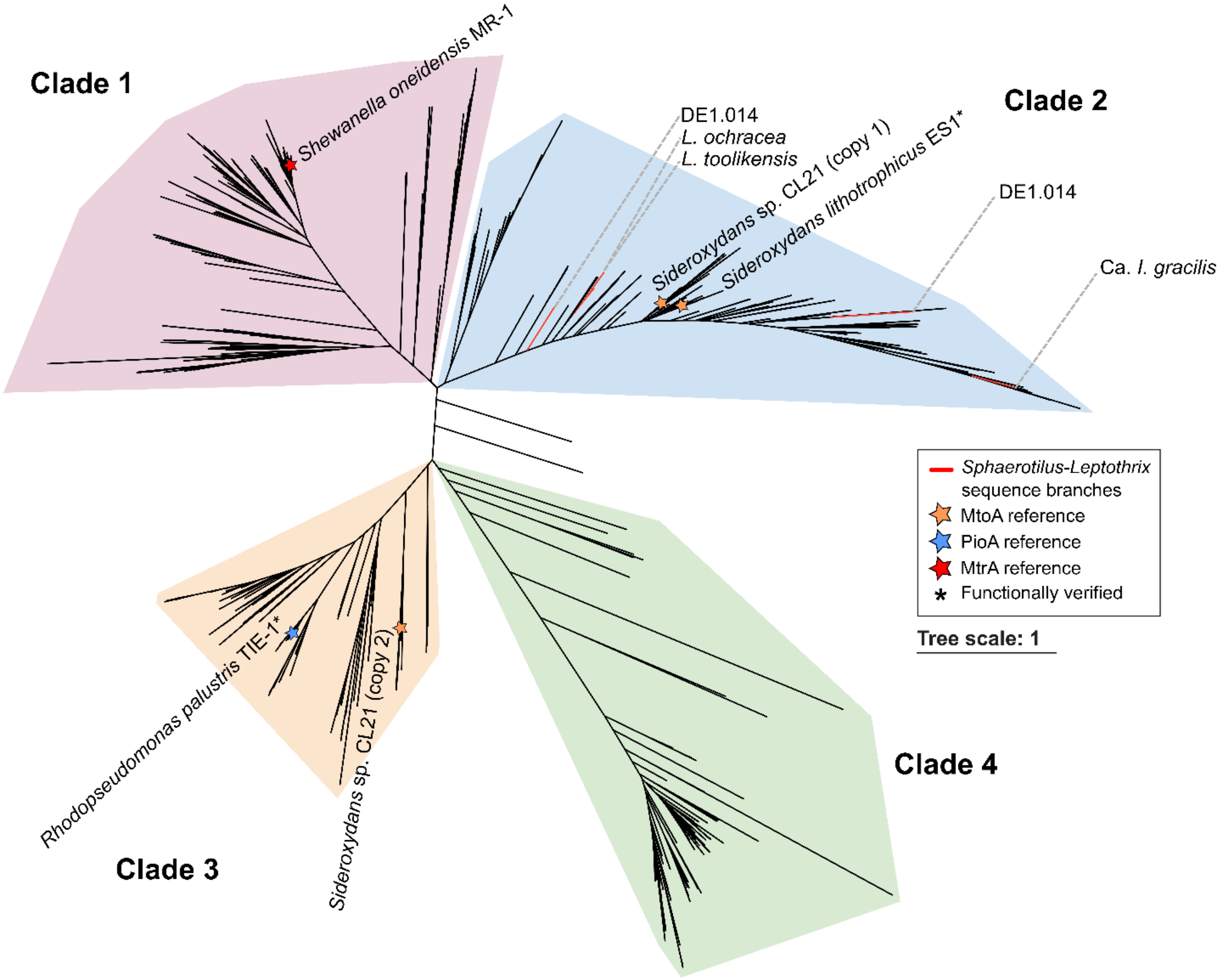

Figure S4. Maximum likelihood tree of MtoA, MtrA, and PioA sequences. Generated using RAxML with 1000 bootstraps. Branches of SLG genomes are shown in red.

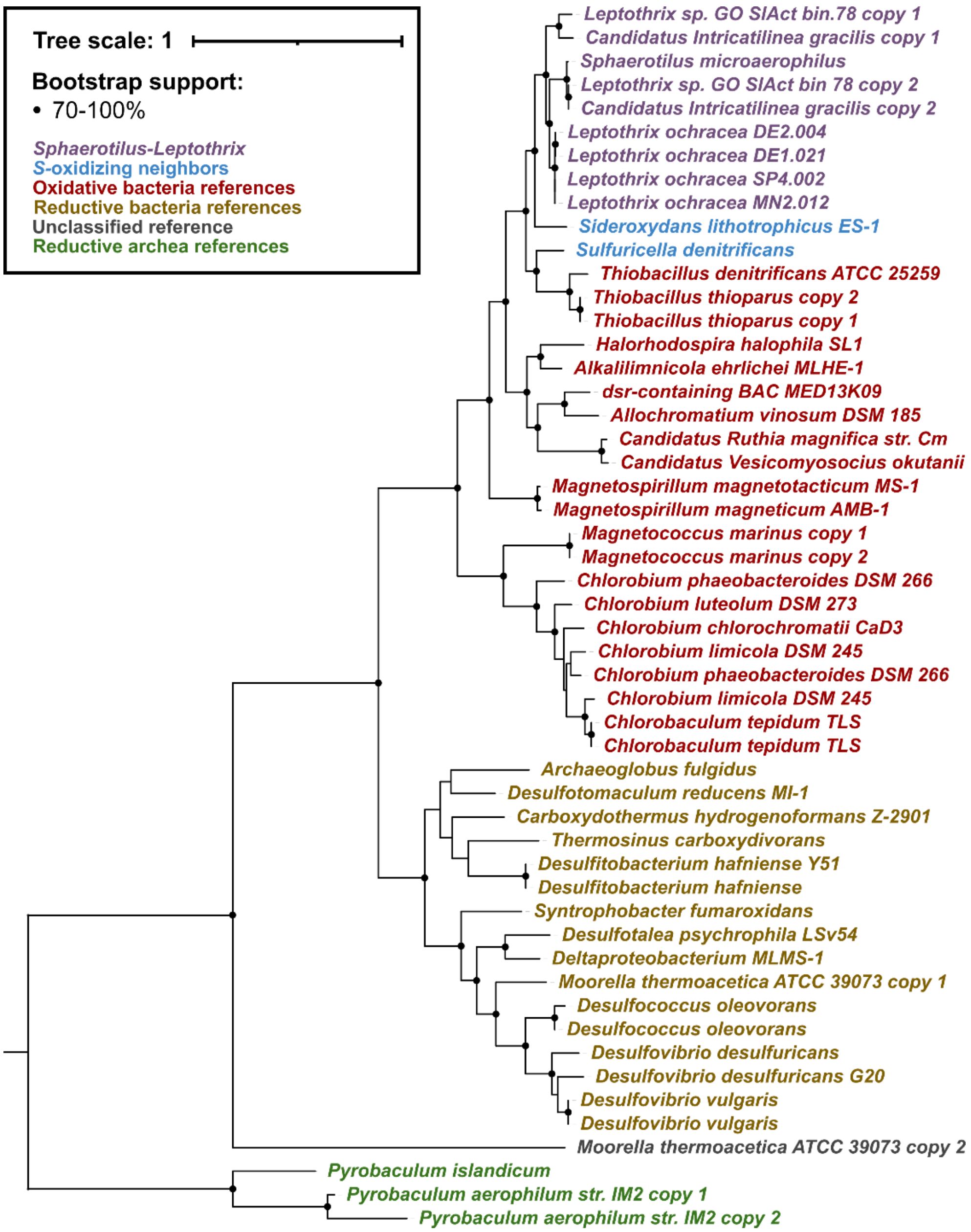

Figure S5. Maximum likelihood tree of DsrA sequences including oxidative and reductive references from Loy et al. (25) and Muller et al. (26). Generated using RAxML with 1000 bootstraps.

SUPPLEMENTAL REFERENCES

1. Emerson D, Ghiorse WC. 1992. Isolation, Cultural Maintenance, and Taxonomy of a Sheath-Forming Strain of *Leptothrix discophora* and Characterization of Manganese-Oxidizing Activity Associated with the Sheath. Appl Environ Microbiol 58:4001–4010.

2. Fomenkov A, Grabovich M, Belousova E, Smolyakov D, Dubinina G, Roberts RJ. 2019. Complete Genome Sequence and Methylome Analysis of *Sphaerotilus natans* subsp. *sulfidivorans* D-507. Microbiol Resour Announc 8:e01194-19.

3. Goeker M. 2016. The One Thousand Microbial Genomes Phase 4 Project (KMG-4) sequencing the most valuable type-strain genomes for metagenomic binning, comparative biology and taxonomic classification. DOE Joint Genome Institute.

4. Grabovich MY, Smolyakov DD, Beletsky AV, Mardanov AV, Gureeva MV, Markov ND, Rudenko TS, Ravin NV. 2021. Reclassification of *Sphaerotilus natans* subsp. *sulfidivorans* Gridneva et al. 2011 as *Sphaerotilus sulfidivorans* sp. nov. and comparative genome analysis of the genus *Sphaerotilus*. Arch Microbiol 203:1595–1599.

5. Gridneva E, Chernousova E, Dubinina G, Akimov V, Kuever J, Detkova E, Grabovich M. 2011. Taxonomic investigation of representatives of the genus *Sphaerotilus*: descriptions of *Sphaerotilus montanus* sp. nov., *Sphaerotilus hippei* sp. nov., *Sphaerotilus natans* subsp. *natans* subsp. nov. and *Sphaerotilus natans* subsp. *sulfidivorans* subsp. nov., and an emended description of the genus *Sphaerotilus*. International Journal of Systematic and Evolutionary Microbiology 61:916–925.

6. Huang K, He Y, Wang W, Jiang R, Zhang Y, Li J, Zhang X-X, Wang D. 2024. Temporal differentiation in the adaptation of functional bacteria to low-temperature stress in partial denitrification and anammox system. Environmental Research 244:117933.

7. Machin EV, Roldán DM, Menes RJ. 2024. *Sphaerotilus uruguayifluvii* sp. nov., a novel filamentous bacterium isolated from river water. Antonie van Leeuwenhoek 117:96.

8. Michoud G, Kohler TJ, Peter H, Brandani J, Busi SB, Battin TJ. 2023. Unexpected functional diversity of stream biofilms within and across proglacial floodplains despite close spatial proximity. Limnology & Oceanography 68:2183–2194.

9. Narihara S, Chida S, Matsunaga N, Akimoto R, Akimoto M, Hagio A, Mori T, Nittami T, Sato M, Mun S, Kang H, Back JH, Takeda M. 2024. Taxonomic characterization of *Sphaerotilus microaerophilus* sp. nov., a sheath-forming microaerophilic bacterium of activated sludge origin. Arch Microbiol 206.

10. Park S, Kim D-H, Lee J-H, Hur H-G. 2014. *Sphaerotilus natans* encrusted with nanoball-shaped Fe(III) oxide minerals formed by nitrate-reducing mixotrophic Fe(II) oxidation. FEMS Microbiol Ecol 90:68–77.

11. Schneider D, Zühlke D, Poehlein A, Riedel K, Daniel R. 2021. Metagenome-Assembled Genome Sequences from Different Wastewater Treatment Stages in Germany. Microbiol Resour Announc 10:e00504-21.

12. Siering PL, Ghiorse WC. 1996. Phylogeny of the *Sphaerotilus-Leptothrix* Group Inferred from Morphological Comparisons, Genomic Fingerprinting, and 16S Ribosomal DNA Sequence Analyses. International Journal of Systematic Bacteriology 46:173–182.

13. Singleton CM, Petriglieri F, Kristensen JM, Kirkegaard RH, Michaelsen TY, Andersen MH, Kondrotaite Z, Karst SM, Dueholm MS, Nielsen PH, Albertsen M. 2021. Connecting structure to function with the recovery of over 1000 high-quality metagenome-assembled genomes from activated sludge using long-read sequencing. Nat Commun 12:2009.

14. Stokes JL. 1954. STUDIES ON THE FILAMENTOUS SHEATHED IRON BACTERIUM *SPHAEROTILUS NATANS*. J Bacteriol 67:278–291.

15. Tee HS, Waite D, Payne L, Middleditch M, Wood S, Handley KM. 2020. Tools for successful proliferation: diverse strategies of nutrient acquisition by a benthic cyanobacterium. The ISME Journal 14:2164–2178.

16. Tothero GK, Hoover RL, Farag IF, Kaplan DI, Weisenhorn P, Emerson D, Chan CS. 2024. *Leptothrix ochracea* genomes reveal potential for mixotrophic growth on Fe(II) and organic carbon. Appl Environ Microbiol 90:e00599-24.

17. Veloso M, Waldisperg A, Arros P, Berríos-Pastén C, Acosta J, Colque H, Varas MA, Allende ML, Orellana LH, Marcoleta AE. 2023. Diversity, Taxonomic Novelty, and Encoded Functions of Salar de Ascotán Microbiota, as Revealed by Metagenome-Assembled Genomes. Microorganisms 11:2819.

18. Ducluzeau A-L, Ouchane S, Nitschke W. 2008. The cbb3 Oxidases Are an Ancient Innovation of the Domain Bacteria. Molecular Biology and Evolution 25:1158–1166.

19. Bosma G, Braster M, Stouthamer AH, Verseveld HW. 1987. Isolation and characterization of ubiquinol oxidase complexes from *Paracoccus denitrificans* cells cultured under various limiting growth conditions in the chemostat. Eur J Biochem 165:657–663.

20. Gabel C, Maier RJ. 1993. Oxygen-dependent transcriptional regulation of cytochrome aa3 in *Bradyrhizobium japonicum*. J Bacteriol 175:128–132.

21. Flory JE, Donohue TJ. 1997. Transcriptional control of several aerobically induced cytochrome structural genes in *Rhodobacter sphaeroides*. Microbiology 143:3101–3110.

22. Van Veen WL, Mulder EG, Deinema MH. 1978. The *Sphaerotilus-Leptothrix* group of bacteria. Microbiol Rev 42:329–356.

23. Spring S. 2006. The Genera *Leptothrix* and *Sphaerotilus*, p. 758–777. *In* Dworkin, M, Falkow, S, Rosenberg, E, Schleifer, K-H, Stackebrandt, E (eds.), The Prokaryotes. Springer New York, New York, NY.

24. Barco RA, Garrity GM, Scott JJ, Amend JP, Nealson KH, Emerson D. 2020. A Genus Definition for *Bacteria* and *Archaea* Based on a Standard Genome Relatedness Index. mBio 11:e02475-19.

25. Loy A, Duller S, Baranyi C, Mußmann M, Ott J, Sharon I, Béjà O, Le Paslier D, Dahl C, Wagner M. 2009. Reverse dissimilatory sulfite reductase as phylogenetic marker for a subgroup of sulfur-oxidizing prokaryotes. Environmental Microbiology 11:289–299.

26. Müller AL, Kjeldsen KU, Rattei T, Pester M, Loy A. 2015. Phylogenetic and environmental diversity of DsrAB-type dissimilatory (bi)sulfite reductases. The ISME Journal 9:1152–1165.
